# Exploring Novel Fungal–Bacterial Consortia for Enhanced Petroleum Hydrocarbon Degradation

**DOI:** 10.3390/toxics12120913

**Published:** 2024-12-17

**Authors:** João Paulo Silva Monteiro, André Felipe da Silva, Rubens Tadeu Delgado Duarte, Admir José Giachini

**Affiliations:** 1 Postgraduate Program in Biotechnology and Biosciences, Department of Microbiology, Immunology and Parasitology, Federal University of Santa Catarina—Campus Reitor João David Ferreira Lima, Florianópolis 88040-900, SC, Brazil; rubens.duarte@ufsc.br (R.T.D.D.); admir.giachini@ufsc.br (A.J.G.); 2 Bioprocess and Biotechnology Engineering Undergraduate Program, Federal University of Tocantins, Gurupi 77402-970, TO, Brazil; andrexfelipe@mail.uft.edu.br

**Keywords:** co-cultures, biodegradation, petroleum hydrocarbons, fungal consortia, bacterial consortia, diesel B20

## Abstract

Bioremediation, involving the strategic use of microorganisms, has proven to be a cost-effective alternative for restoring areas impacted by persistent contaminants such as polycyclic aromatic hydrocarbons (PAHs). In this context, the aim of this study was to explore hydrocarbon-degrading microbial consortia by prospecting native species from soils contaminated with blends of diesel and biodiesel (20% biodiesel/80% diesel). After enrichment in a minimal medium containing diesel oil as the sole carbon source and based on 16S rRNA, Calmodulin and β-tubulin gene sequencing, seven fungi and 12 bacteria were identified. The drop collapse test indicated that all fungal and four bacterial strains were capable of producing biosurfactants with a surface tension reduction of ≥20%. Quantitative analysis of extracellular laccase production revealed superior enzyme activity among the bacterial strains, particularly for *Stenotrophomonas maltophilia* P05R11. Following antagonistic testing, four compatible consortia were formulated. The degradation analysis of PAHs and TPH (C5–C40) present in diesel oil revealed a significantly higher degradation capacity for the consortia compared to isolated strains. The best results were observed for a mixed bacterial-fungal consortium, composed of *Trichoderma koningiopsis* P05R2, *Serratia marcescens* P10R19 and *Burkholderia cepacia* P05R9, with a degradation spectrum of ≥91% for all eleven PAHs analyzed, removing 93.61% of total PAHs, and 93.52% of TPH (C5–C40). Furthermore, this study presents the first report of *T. koningiopsis* as a candidate for bioremediation of petroleum hydrocarbons.

## 1. Introduction

Anthropogenic activities involving the supply chain of petroleum-based products are frequently associated with accidental spills into the environment [1,2]. As a result, the exposure to such substances can cause irreversible impacts on the organisms and ecosystems affected [3]. In its composition, crude oil comprises a complex mixture of hydrocarbons (polycyclic aromatic, monoaromatic, aliphatic, and cycloalkanes), asphaltenes, and resins [4]. Among these constituents, polycyclic aromatic hydrocarbons (PAHs) are recognized by their persistence in the environment, attributed to physicochemical properties like low solubility, high molecular weight, and stability resulting from the fusion of two or more aromatic rings [5,6]. Furthermore, the bioaccumulation capacity of these compounds is associated with cytotoxic, carcinogenic, mutagenic, and teratogenic effects [7,8,9]. Consequently, the accidental release of these contaminants and their long-term effects are of great concern, emphasizing the need for alternative energy sources as well as innovative approaches focused on environmental remediation and restoration [6,10].

The use of biofuels derived from renewable biomass offers an alternative to expanding the global energy matrix [11]. Among the biofuels utilized in the transportation sector, biodiesel oil stands out for its capacity to replace mineral-derived diesel oil or be incorporated into binary blends composed of diesel/biodiesel [12]. In Brazil, the production and demand for biodiesel oil have been increasing through public policies, which currently mandate the inclusion of 14% (*v*/*v*) biodiesel in commercial diesel, forming the B14 blend [13,14]. On the other hand, despite biodiesel being described as a biodegradable fuel with low toxicity [15,16], mineral diesel oil, which constitutes the largest portion of diesel/biodiesel binary blends, consists of a complex mixture of hydrocarbons known for their harmful effects [17], posing a risk in the face of accidental releases associated with the increased usage of these blends.

In response to this environmental challenge, sustainable remediation technologies have been receiving increasing attention [18,19]. From this perspective, microbial bioremediation has demonstrated significant advantages over physico-chemical techniques that are generally associated with complex pre-treatments, inefficient removal of residual contaminants, and the high overall cost of the process [20]. This efficient, low-cost biological system can mediate the biotransformation, detoxification, and permanent removal of a wide range of organic contaminants, including high molecular weight PAHs [10,21]. However, in order to establish a system that promotes the consumption and mineralization of contaminants, the selection of microorganisms equipped with appropriate metabolic mechanisms is essential [22].

As a source for bioprospecting, environments impacted by petroleum hydrocarbons have shown a strong influence on the modulation of native microbial communities [23,24]. These findings are supported by studies revealing numerous hydrocarbon-degrading fungal genera in contaminated areas, such as *Aspergillus*, *Penicillium*, *Pleurotus*, *Trametes*, *Fusarium,* and *Trichoderma* [25,26,27,28], along with some bacteria like *Alcanivorax*, *Pseudomonas*, *Bacillus*, *Rhodococcus*, *Sphingomonas*, *Burkholderia*, *Paraburkholderia*, *Serratia,* and *Stenotrophomonas* [29,30,31]. Among the members of this microbial community, several fungal species are recognized for their secretion of important oxidative enzymes, such as laccases (LaC, EC:1.10. 3.2), manganese peroxidases (MnP, EC:1.11.1.13), lignin peroxidases (LiP, EC:1.11.1), and versatile peroxidases (VP, EC:1.11.1.16) [32]. These enzymes act nonspecifically, catalyzing the simultaneous oxidation of a large number of aromatic and non-phenolic compounds, with the potential to degrade different types of hydrocarbons [19,33]. For example, the ability of a *Penicillium oxalicum* strain to produce LaC, MnP, and VP has demonstrated a direct correlation with increased degradation of high molecular weight PAHs, including anthracene, dibenzothiophene, phenanthrene, and dibenzofuran [34]. Similarly, Pozdnyakova et al. [35] reported that the combined activity of LaC and VP expressed by a *Pleurotus ostreatus* strain was responsible for converting phenanthrene and anthracene into quinones that integrated into the basal metabolism, leading to the mineralization of these compounds.

Beyond these fungal mechanisms, numerous bacterial species are recognized for their significant role in PAH degradation, employing mixed-function oxygenase enzymes, such as monooxygenases, dioxygenases, peroxidases, hydroxylases, and dehydrogenases, to efficiently metabolize these compounds [36]. For example, the combined activity of enzymes like alkane hydroxylase, alcohol dehydrogenase, and laccases, produced by *Pseudomonas stutzeri* NA3 and *Acinetobacter baumannii* MN3, have been shown to enhance crude oil degradation, achieving removal efficiencies of 84% and 78%, respectively [37]. In another study, a functional analysis of *Delftia* sp. and *Burkholderia* sp. during phenanthrene degradation revealed the ‘phn’ genomic island as an integrative element containing key genes for PAH degradation, including phenanthrene dioxygenase, which catalyzes the initial oxidation of the aromatic ring. The presence of hydrocarbons in the medium upregulated *phn* genes, directly linking their expression to PAHs exposure. Additionally, proteomic profiling revealed increased expression of proteins like *PhnF* and *PhnJ*, along with downstream catabolic enzymes, underscoring these bacteria’s adaptive mechanisms in PAH-contaminated environments [38].

Another important metabolic strategy known to enhance the degradation of hydrocarbons is the production of biosurfactants by certain species of fungi and bacteria [39,40]. These active surface molecules reduce the interfacial and surface tension, increasing the contact surface area of the hydrophobic portions of contaminants, which leads to enhanced bioavailability for subsequent biodegradation [41,42]. According to Mnif et al. [43], a proportional relationship between the production of biosurfactants by a *Bacillus subtilis* strain and the biodegradation of diesel oil and kerosene seems to be the rule of thumb. Combined with these mechanisms, mutualistic associations between bacteria and fungi have demonstrated a critical role in the biodegradation of complex mixtures of hydrocarbons [44].

Since a single microorganism typically metabolizes only a limited range of hydrocarbons, synergism through complementary biochemical pathways enables one organism to degrade a metabolite produced by another, in order to complete the hydrocarbon degradation process or alleviate potential toxic/inhibitory effects [45,46].

In this context, bioremediation through the use of mixed cultures of fungi and bacteria equipped with different metabolic mechanisms can enhance the extent and efficacy of biodegradation [47,48]. Atapka et al. [49], for example, suggest that combining fungal strains with biosurfactant-producing bacteria effectively improves the degradation of petroleum hydrocarbons. According to the authors, the use of a consortium composed by *Acinetobacter* sp. and *Scedosporium* sp. provided higher rates of total petroleum hydrocarbon (TPH) degradation than when the strains were analyzed individually. In another study, Ameen et al. [50] demonstrated that a consortium composed of *Alternaria alternata*, *Aspergillus terreus*, *Cladosporium sphaerospermum*, *Eupenicillium hirayamae*, and *Paecilomyces variotii*, isolated from hydrocarbon-contaminated mangrove sediments, was able to completely degrade the main hydrocarbons present in diesel oil. As observed, the synergistic effect of the combined genotypes for the production of complex enzymes (Lac, LiP, and MnP) was significantly higher compared to the strains evaluated alone.

Aligned with this information, the primary objective of this study was to develop microbial consortia with potential for application in the bioremediation of petroleum hydrocarbons. To achieve this goal, fungal and bacterial strains were isolated from experimental areas intentionally contaminated with B20 biodiesel (20% *v*/*v* biodiesel and 80% *v*/*v* diesel). After identifying the isolates, different combinations of microbial consortia were designed based on the results for the screening of extracellular laccases, biosurfactants, and species compatibility. These novel consortia and individual strains, including the *Trichoderma koningiopsis* strain evaluated in this context for the first time, were subsequently tested for their diesel oil degradation potential. The results highlighted the effectiveness of these microbial partnerships in targeting and degrading complex hydrocarbon pollutants, suggesting promising applications in environmental bioremediation.

## 2. Materials and Methods

### 2.1. Collecting Area

The samples utilized in the present study were collected at the Ressacada Experimental Farm, in Florianópolis, southern Brazil (latitude 27°30′ S, longitude 48°30′ W), where controlled contamination procedures with fuels and biofuels have been monitored for 25 years (1998–2023) through research projects linked to PETROBRAS in partnership with the Ressacada Environmental Research Center (REMA) and the Federal University of Santa Catarina (UFSC). Based on historical analytical data, the experimental release areas P05 (B100-ANM) and P10 (B20-BAF) were selected to prospect petroleum hydrocarbon-degrading microorganisms and analyze the current concentration of contaminating hydrocarbons. The experimental area P05 (B100-ANM) was contaminated with 100 L of B20 biodiesel (20% *v*/*v* soybean biodiesel and 80% *v*/*v* diesel), which had been subjected to natural attenuation remediation starting in 2008. Area P10, contaminated with 100 L of B20 biodiesel (20% *v*/*v* palm biodiesel and 80% *v*/*v* diesel), underwent remediation through bioaugmentation combined with Fe_2_O_3_ in 2017 [51].

Soil samples from the selected areas were collected on April 28, 2021, using a Dutch auger. With the aid of the equipment, three perforations were made at points near the contamination source well. According to previous data on the depth of contaminant release in the soil, samples were collected at 1.5 m depth for area P05 and 1.8 m depth for area P10. After collection, mixed soil compositions were prepared by homogenizing the samples. Approximately 500 g of soil kept at a temperature of 4 °C were sent to the laboratory for analysis.

### 2.2. Evaluation of PAHs and BTEX Levels at Sampling Sites

Employing the EPA 3550C method with some modifications, approximately 4 g of dry, homogenized soil was transferred to 20 mL vials for the extraction of PAHs [52]. Dichloromethane (CH_2_Cl_2_) was chosen as the solvent for ultrasonic extraction and the process was performed under a Cole-Parmer 8890E-MTH ultrasonic device at a frequency of 55 Hz for 30 min. This process was repeated three times, using 6 mL of solvent per step. After extraction, the organic phase extracts were allowed to settle for 10 min to facilitate phase separation. The extracts were then vacuum filtered using PTFE membranes with a pore size ≤ 0.45 μm. The final extract was concentrated under a gentle stream of nitrogen gas and transferred to vials. PAH concentrations were evaluated using an HP 6890 Series II gas chromatograph with a Headspace auto-sampler G1888, a flame ionization detector (FID), and an HP5 capillary column (J&W Scientific, Agilent Technologies, Santa Clara, CA, USA). Helium served as the carrier gas at a flow rate of 3.0 mL·min^−1^. The injector and detector temperatures were set at 260 °C and 320 °C, respectively. The oven temperature was programmed as follows: an initial ramp from 60 °C to 200 °C at a rate of 4 °C min^−1^, followed by a final ramp from 200 °C to 280 °C at a rate of 2 °C min^−1^.

For the extraction of BTEX (benzene, toluene, ethylbenzene, and xylenes), approximately 1 g of soil was weighed into 20 mL vials, and the volume was adjusted to 10 mL with ultrapure water, following the EPA 5021A method combined with EPA 8015D [53,54]. Quantification was performed using a gas chromatograph with an HP1 column. Helium was again used as the carrier gas at a flow rate of 3.0 mL·min^−1^. The injector and detector temperature were set at 300 °C and the oven temperature was ramped from 70 °C to 120 °C at a rate of 6 °C·min^−1^. The detection limits for each individual compound are detailed in other sources [55,56].

### 2.3. Enrichment and Isolation of Hydrocarbon-Degrading Strains

The isolation and selection of fungal and bacterial species with potential for biodegrading petroleum hydrocarbons were conducted using the enrichment technique in minimal medium with some modifications [57]. Initially, 5 g of soil samples from the contaminated areas were transferred to 250 mL Erlenmeyer flasks containing 80 mL of Bushnell Haas—BH medium—composed of (g/L): 1 g K_2_HPO_4_, 0.2 g MgSO_4_, 1 g KH_2_PO_4_, 0.02 g CaCl_2_, 1 g NH_4_NO_3_, 0.05 g FeCl_2_ [58], enriched with 1% (*v*/*v*) filtered commercial diesel oil as the only carbon source, which was added after sterilization. The flasks were maintained on an orbital shaker (150 rpm, 28 °C), with samples collected at the following two distinct periods: at 7 and 14 days after incubation. The enriched cultures were then serially diluted (10^−6^ to 10^−9^) and spread over sterile plates with Nutrient Agar—NA (BD Difco, Franklin Lakes, NJ, USA)—and Malt Extract Agar—MEA (Millipore, Burlington, NJ, USA)—and incubated at 28 °C ± 2 °C for 7 days. Resistive colonies, selected based on visual differences in morphology, size, and coloration, were re-streaked to obtain pure colonies. The axenic cultures were then subjected to a second enrichment procedure in BH mineral medium enriched with 5% (*v*/*v*) diesel oil. After the incubation period, bacterial isolates that still exhibited viable colonies were further assessed for cell wall type, morphology, and arrangement using Gram staining [59]. Micromorphology of fungal isolates was assessed through the microculture technique [60]. To observe additional taxonomic features, such as colony color, texture, production of soluble pigments and exudates, the fungal isolates were point-inoculated onto sterile plates containing Czapek Yeast Extract Agar—CYA [61]—Yeast Extract Sucrose Agar—YES [62]—and MEA. The plates were then incubated at 28 °C for 7 days.

### 2.4. Identification of Hydrocarbon-Degrading Fungi and Bacteria

Total genomic DNA from the isolated fungi and bacteria was extracted using the Plant/Fungi DNA Isolation Kit (Norgen Biotek, Thorold, ON, Canada) and the DNeasy Kit (Qiagen, Hilden, Germany), respectively. DNA concentration and purity were assessed using a NanoVue Plus spectrophotometer (GE). For fungal species identification, the genomic DNA was subjected to amplification of partial regions of the β-tubulin gene (BenA) using the primers Bt2a (5′-GGT AAC CAA ATC GGT GCT GCT TTC-3′) and Bt2b (5′-ACC CTC AGT GTA GTG ACC CTT GGC-3′) [63]. Additionally, partial regions encoding the Calmodulin (CaM) gene were amplified using primers CMD5 (5′-CCG AGT ACA AGG AGG CCT TC-3′) and CMD6 (5′-CCG ATA GAG GTC ATA ACG TGG-3′) [64]. These genes were selected for their ability to provide higher resolution in distinguishing closely related fungal species, which is particularly useful for species within the *Aspergillus* and *Penicillium* genera [65].

Genomic DNA extracted from isolated bacteria was amplified using primers 27F (-AGA GTT TGA TCM TGG CTC AG-) [66] and 1492R (-GGT TAC CTT GTT ACG ACT T) [67] to obtain 16S rRNA gene fragments. PCR products were visualized on a 1% agarose gel stained with GelRed (Invitrogen, Carlsbad, CA, USA). The amplicons were purified using the PCR Products Purification Kit (Ludwig, Alvorada, Brazil) and sent for Sanger sequencing at ACTGene Company (Brazil). The sequences were submitted to the GenBank database to align with published sequences using NCBI BLASTN. The nucleotide sequences of isolates were deposited in GenBank and accession numbers were obtained.

### 2.5. Phylogenetic Analysis

A Kimura 2-parameter phylogenetic tree (K2 + G) was constructed with the MEGA 7 software version 7.0 [68], based on the maximum likelihood (ML) test for fungal sequences and the Neighbor-Joining (NJ) test for bacterial sequences, with 1000 bootstraps [69,70]. In addition to 16S rRNA gene amplification, bacterial isolates were further differentiated by comparing the repetitive DNA banding profiles generated using the BOX-PCR technique [71]. Genomic DNA was used as the template for amplification, following the procedure described by Versalovic et al. [71] and using the primer A1R (5′-CTA CGG CAA GGC GAC GCT GAC G-3′). A control reaction was performed by substituting genomic DNA with ultrapure water. After the amplification reaction, the products were visualized through electrophoresis on a 1.2% (*w*/*v*) agarose gel, run at a voltage of 3 volts per centimeter. The gel image was photographed using a UV transilluminator and the ChemiDoc MP Digital Imaging System (Bio-Rad, Hercules, CA, USA). Bands were clustered based on similarity, and a dendrogram was constructed using the UPGMA method and Jaccard coefficient in software NTSYS version 2.1 [72,73].

### 2.6. Screening for Biosurfactants

To evaluate the potential production of biosurfactants, twelve bacterial strains were initially cultivated for 48 h (130 rpm, 30 °C) in 250 mL Erlenmeyer flasks containing 50 mL of nutrient broth (BD Difco). Subsequently, 1% *v*/*v* of the inoculum was transferred to Erlenmeyer flasks containing 50 mL of Mineral Salts Medium—MSM—which consists of (g/L): 7.6 g Na_2_HPO_4_, 4.43 g KH_2_PO_4_, 6 g (NH_4_)_2_SO_4_, 0.4 g MgSO_4_, 0.4 g CaCl_2_, and 2 mL of a trace element solution (20.1 g disodium EDTA, 16 g FeCl_3_·6H_2_O, 0.18 g CoCl_2_·6H_2_O, 0.18 g ZnSO_4_·7H_2_O, and 0.16 g CuSO_4_·5H_2_O) [74]. The medium was supplemented with 3% (*v*/*v*) filtered soybean oil, which was added after sterilization. The flasks were incubated in an orbital shaker (130 rpm, 30 °C) for 7 days. After incubation, the total volume of each culture was transferred to 50 mL Falcon tubes and centrifuged at 4.500 rpm for 15 min. The supernatant was then used for subsequent tests.

The fungal species were initially cultivated on MEA for 4 to 7 days at 28 °C. Two mycelial disks (5 mm) were then transferred to 250 mL Erlenmeyer flasks containing 50 mL of medium composed of (g/L): 3 g KH_2_PO_4_, 5 g NaNO_3_, 3 g MgSO_4_·7H_2_O, 3 g FeSO_4_·7H_2_O, 0.01 g MnSO_4_·H_2_O, 0.01 g ZnSO_4_, and 0.2% (*w*/*v*) yeast extract [75]. The medium was supplemented with 3% (*v*/*v*) soybean oil, added after sterilization. The cultures were incubated on an orbital shaker (120 rpm, 28 °C) for 7 days. After the incubation period, the total volume of each culture was transferred to 50 mL Falcon tubes and were subjected to ultrasonic baths at room temperature for 5 min, to release biosurfactants adhered to the biomass and cell membrane. The samples were then centrifuged at 4.500 rpm for 15 min, and the supernatant was used to evaluate biosurfactant production via the pendent-drop method [76,77]. The pendent-drop assay is based on the destabilization of liquid oil droplets by surfactants. The surface tension at the water–oil interface causes an oil droplet to be repelled by a hydrophilic surface. However, when the liquid on the surface contains surfactants, they reduce the surface tension, causing the droplet to spread or collapse [40,78]. The biosurfactant production cultures were prepared in triplicate, with non-inoculated flasks serving as controls.

The determination of surface tension was conducted using approximately 3 mL of culture supernatant. Surface tension measurements (in mN·m^−1^) were performed using a goniometer 250-F1 (Ramé-Hart Instrument Co., Succasunna, NJ, USA) (Supplementary Figure S1). The goniometer was calibrated using distilled water, and the syringe used was rinsed three times with distilled water and twice with the sample to be analyzed. The DROPimage Advanced software was employed to analyze the curvature profile of the pendant drop formed at the tip of the needle. Ten measurements were taken at one second intervals for each reading, and the final result was the average of these ten measurements. All measurements were performed in triplicate at room temperature (25 °C to 28 °C). Furthermore, the percentage of surface tension reduction (STR) was calculated to assess the potential production of biosurfactants. The calculation was derived from the ratio between the negative variation in surface tension during cultivation and the surface tension of the negative control, multiplied by 100 [40].

### 2.7. Screening for Laccases

In a preliminary screening step, all isolated fungal and bacterial strains were assessed for qualitative laccase activity to identify those with the potential for further quantitative analysis. Following the methodology adapted from Karp et al. [79], mycelial disks from seven isolated fungal strains were used as inoculum. Two disks were transferred to 250 mL Erlenmeyer flasks containing 80 mL of modified Czapek Yeast Extract broth (g/L): 10 mL of concentrated Czapek solution [30 g NaNO_3_, 5 g KCl, 5 g MgSO_4_·7H_2_O, 0.1 g FeSO_4_·7H_2_O, 0.1 g ZnSO_4_·7H_2_O, and 100 mL of distilled water], 1 g K_2_HPO_4_, 2.5 g yeast extract, and 15 g dextrose. The medium was supplemented with 150 μM CuSO_4_·5H_2_O, which was pre-filtered and added after sterilization. The flasks were incubated on an orbital shaker (120 rpm, 28 °C) for 12 days, with 1 mL aliquots collected every 48 h.

For bacterial species, a pre-culture in nutrient broth was prepared for the twelve selected strains. An aliquot of 1% *v*/*v* of the pre-culture was transferred to 250 mL Erlenmeyer flasks containing 50 mL of modified MSM broth containing the following (g/L): 2.5 g yeast extract, 1.2 g dextrose, 7 g NaCl, 4 g KH_2_PO_4_, 8 g K_2_HPO_4_, and 0.18 g MgSO_4_, pH 7 [80]. The medium was supplemented with 500 μM CuSO_4_·5H_2_O, pre-filtered, and added after sterilization. The flasks were incubated on an orbital shaker (130 rpm, 30 °C) for 96 h, with 1 mL aliquots collected every 24 h.

The presence of extracellular laccases was assessed using the substrate ABTS-2,2′-azino-bis[3-ethylbenzothiazoline-6-sulfonic acid] (Merck, Rahway, NJ, USA). The enzymatic reaction was prepared for a final volume of 1 mL, composed of the following: 800 μL of 100 mM sodium acetate buffer (pH 4.5), 100 μL of 20 mM ABTS (in distilled water), and 100 μL of crude enzyme extract obtained by centrifuging the collected aliquots at 13.000 rpm for 5 min. The tubes were mixed by inversion and incubated in a dry bath EchoTherm (Torrey Pines Scientific, Carlsbad, CA, USA) for 20 min at 40 °C. After incubation, microtubes (1.5 mL) exhibiting enzymatic activity showed the development of a green–bluish coloration, indicating substrate oxidation. A control reaction was performed by replacing the crude enzyme extract with 100 μL of the control culture medium. All assays were conducted in triplicate.

### 2.8. Growth Conditions for Quantitative Evaluation of Laccases

The species demonstrating the highest levels of oxidation in microtube assays were subsequently monitored under submerged cultivation to determine the levels of extracellular laccase activity. For the selected fungal species, cultivation was performed in 250 mL Erlenmeyer flasks containing 100 mL of modified Czapek Yeast Extract broth, as previously described. Once inoculated, the cultures were maintained on an orbital shaker (110 rpm, 28 °C) for 14 days, with samples collected every 24 h to determine extracellular laccase activity. Bacterial cultivation was carried out in 250 mL Erlenmeyer flasks containing 80 mL of the modified MSM broth. The flasks, inoculated with 1% *v*/*v* of a pre-inoculum, were incubated on an orbital shaker (130 rpm, 30 °C) for 6 days, with samples collected every 24 h to determine extracellular laccase activity and microbial growth by measuring the optical density (OD) of the cultures at 600 nm using a spectrophotometer DR3900 (HACH, Loveland, CO, USA).

### 2.9. Measurement of Extracellular Laccase Activity

The activity of extracellular laccases was assessed by monitoring the oxidation of the ABTS substrate. The reaction mixture consisted of 800 μL of 50 mM sodium acetate buffer (pH 4.5), 100 μL of 10 mM ABTS (in distilled water), and 100 μL of crude enzyme extract obtained by centrifuging the collected aliquots at 13.000 rpm for 5 min [81]. Formation of the ABTS^+^ radical cation was tracked by measuring an absorbance increase at 420 nm using a spectrophotometer. Readings were taken every minute over a 5 min period at room temperature. The control sample consisted of the respective culture media without inoculum. One unit (U) of laccase activity was defined as the amount of enzyme that oxidizes 1 µmol of ABTS per minute at 25 °C, using a molar extinction coefficient for the ABTS^+^ radical cation (ε420 nm = 36,000 M^−1^ cm^−1^) [82]. All assays were conducted in triplicate. Laccase activity was determined based on the formula described by Leonowicz and Grzywnowicz [83], and expressed in U/mL:

$$(U/mL)=\frac{∆Abs\times V\times 10^{6}}{\varepsilon \times Ve\times ∆t}$$

where ∆Abs represents the difference in absorbance values obtained; V is the total sample volume (mL); 10^6^ is the factor used to convert moles to micromoles (μmoles); ε is the molar extinction coefficient for the ABTS^+^ radical cation; Ve is the volume (mL) of crude enzyme extract used; and ∆t is the reaction time (min).

### 2.10. Selection and Assembly of Microbial Consortia

Considering the results of the biosurfactant and laccase assays, six bacterial strains (P05R8; P05R9; P05R11; P05R12; P10R13; P10R19) and five fungal strains (P05R1; R05R2; P05R3; P10R5; P10R7) were selected for analysis of growth dynamics when co-cultivated. This step aimed to formulate microbial consortia capable of degrading petroleum hydrocarbons.

To ensure the stability of the formed consortia, potential antagonistic behavior among the different strains was evaluated by confrontation on sterile plates containing Toyama’s medium composed by the following (g/L): 1 g (NH_4_)_2_SO_4_, 0.5 g MgSO_4_·7H_2_O, 3 g KH_2_PO_4_, 0.5 g NaCl, 0.001 g FeSO_4_, and 10 g dextrose [84], supplemented with 1% *v*/*v* diesel oil, added after autoclaving. Microbial confrontations occurred in the following order: (fungus vs. fungus; bacteria vs. bacteria; bacteria vs. fungus). For the fungus vs. fungus antagonism assay, two species per plate were point-inoculated approximately 2 cm apart. The plates were incubated at 28 °C until inhibition halos or antagonistic effects between fungal colonies were observed. Antagonism between bacteria was evaluated by inoculating two strains per plate following a grid pattern, allowing direct contact between the strains. The plates were incubated at 30 °C until antagonistic effects between the colonies were observed. In the bacteria vs. fungus antagonism assay, fungi were inoculated individually at the center of the plates and then incubated at 28 °C for 4 days. After partial development of the fungal colonies, six bacterial strains were transferred to the periphery of the plates, streaking towards the center of the fungal colonies. The plates were re-incubated at 28 °C until antagonistic effects between the colonies were observed [85]. Based on the compatibility of the strains, consortia of mixed cultures of bacteria, fungi, and bacteria-fungi were formulated.

### 2.11. Biodegradation of Diesel Oil by Individual Strains and Microbial Consortia

The ability to biodegrade diesel oil and its hydrocarbons (PAHs and TPH) was evaluated in 250 mL Erlenmeyer flasks containing 80 mL of BH medium and 1% (*v*/*v*) diesel oil [46]. Both mixed and individual strains were assessed, including the mixed bacteria–fungi consortium FFB1 (P05R2, P05R3, and P10R19), bacterial consortium BB1 (P05R11 and P10R19), fungal consortium FF1 (P05R2 and P05R3), mixed bacteria–fungi consortium FBB1 (P05R2, P10R19, and P05R9), individual fungal strain P05R2, individual fungal strain P05R3, individual bacterial strain P10R19, and individual bacterial strain P05R9.

Initially, for the formulation of the consortia, fungi and bacteria were cultivated individually. Bacterial strains were inoculated into 250 mL Erlenmeyer flasks containing 80 mL of nutrient broth. The flasks were incubated in an orbital shaker (120 rpm, 28 °C) for 24 h. Subsequently, the cells were harvested by centrifugation at 12.000 rpm for 5 min, washed twice in sterile 0.9% NaCl solution, and resuspended in liquid BH medium [86]. The cell suspension concentration was adjusted to an optical density of 1 at 600 nm using a spectrophotometer. Cell density was estimated by plate count technique on NA agar, using serial dilutions (10^−3^ to 10^−7^). Finally, bacterial consortia were prepared by combining the cultures in a 1:1 volumetric ratio [87]. The prepared consortia and the previously mentioned individual bacterial cultures were inoculated at a 1.2% ratio into the flasks containing BH medium supplemented with diesel [49]. Fungal strains were individually inoculated into plates containing MEA and incubated for 7 days at 28 °C. Subsequently, consortia were prepared using two mycelial disks (5 mm) per strain, taken from the edges of the colonies [26]. The disks of individual fungal strains and fungal consortia were transferred to the flasks containing BH medium supplemented with diesel. For flasks containing mixed fungi–bacteria consortia (FFB1 and FBB1), the sequential inoculation method described by Ghorbannezhad, Moghimi, and Dastgheib [46] was adopted, where bacterial strains were added to the BH liquid medium five days after the addition of the fungal strains. The flasks were incubated in an orbital shaker (130 rpm, 28 °C) for 15 days. Abiotic control experiments were prepared by incubating diesel oil in BH medium without inoculum. All assays were performed in triplicate. After the incubation period, samples were collected to assess the viability of the consortia and to determine potential antagonistic interactions. Aliquots were serially diluted (10^−6^ to 10^−9^) and spread onto sterile plates with NA and MEA and incubated at 28 °C ± 2 °C for 7 days. Subsequently, the cell biomass was removed from the culture medium by centrifugation at 7000 rpm for 15 min. The remaining hydrocarbons in the supernatant were extracted using solid phase (SPE) cartridges, according to the EPA 525.2 method [88], and measured by gas chromatography. The efficacy of the consortia and individual strains was expressed as a percentage of degradation (%) for the hydrocarbons (PAHs and TPH). This was calculated based on the difference between the average concentrations in the control samples and the average concentrations in the inoculated samples, multiplied by 100.

### 2.12. Statistical Analyses

Statistically significant differences among the experimental treatments were analyzed using analysis of variance (ANOVA) followed by the Scott–Knott multiple comparison test at a 0.05 significance level. Homogeneity of variance was assessed using Bartlett’s test, and normality of residuals was verified using the Shapiro–Wilk test [89,90].

## 3. Results

### 3.1. Evaluation of PAHs and BTEX Levels at Sampling Sites

Surprisingly, even a decade after the release of these contaminants in area P05 (2008), the current study revealed residual concentrations of numerous PAHs and significantly elevated levels of BTEX (Table 1). For more precise comparison parameters, we utilized the definitions provided by the National Environment Council (CONAMA, Brazil), which, through Resolution No. 420/2009, establishes guideline values for soil contamination prevention and sets concentration limits for these substances. The analysis of samples from this area indicated that monoaromatic hydrocarbons such as benzene, toluene, and xylenes had concentrations exceeding the recommended limits for soil. Among the PAHs, only naphthalene and benzo(a)anthracene were detected in concentrations above the established limits. However, it is important to note that reference values for some types of PAHs are not covered by the regulatory agency’s guidelines, as presented in Table 1. In area P10, contaminated in 2017, concentrations above the recommended average were detected for xylenes, toluene, naphthalene, and anthracene.

### 3.2. Enrichment and Isolation of Hydrocarbon-Degrading Microbial Strains

A total of 19 isolates exhibited viable colonies after two enrichment stages in minimal mineral medium containing commercial diesel oil as the sole carbon source. Of these isolates, 8 originated from area P05, while 11 were from area P10. They were designated using the following nomenclature: P (05 or 10) + R (REMA) + Nº (from 1 to 19). Macro- and micromorphological analyses initially segregated them into two distinct groups, comprising seven fungi and 12 bacteria. Among the fungal isolates, contrasting morphotypes were observed in MEA, CYA, and YES culture media (Supplementary Figure S2). Isolates P05R1, P05R3, P10R4, P10R5, P10R6, and P10R7 exhibited velvety textured colonies with radial fissures, grooves on the reverse side, and colors ranging from white, cream, orange, to predominantly green. The presence of septate hyphae with conidiophores (either monoverticillate or biverticillate) and short phialides supporting chains of conidia indicated that these isolates were consistent with the genus *Penicillium* [65], likely representing multiple species and/or strains of the genus. In contrast, isolate P05R2 maintained consistent characteristics across different media, presenting white filamentous cotton-like colonies with rapidly developing aerial mycelium (2 to 4 days), unlike the first group of fungi (7 to 10 days depending on the medium). Micromorphological analyses identified the presence of septate hyphae and smooth globose conidia. Based on the information provided by the study of Samuels et al. [91], the isolate exhibited similarities with species of the genus *Trichoderma.* Regarding the 12 bacterial isolates, colonies ranged from white to yellow with various textures (opaque, shiny, translucent, and gelatinous). Approximately 25% of the isolates were Gram-positive, while 75% stained as Gram-negative.

### 3.3. Identification of Hydrocarbon-Degrading Fungi and Bacteria

The isolated fungi belonged to the phylum *Ascomycota*, confirming the results of macro- and micromorphological analyses. Comparison of partial sequences of the β-tubulin and Calmodulin genes with those available in the NCBI GenBank revealed six species in the genus *Penicillium* and one species in the genus *Trichoderma* (Table 2).

According to the collecting areas, some distinct species were identified in area P10 (*P. janthinellum* and *T. koningiopsis*) and P05 (*Penicillium* sp. and *Penicillium pulvillorum*). The phylogenetic tree, constructed from the alignment of *BenA* and *CaM* gene sequences for the reference strains of the *Penicillium* section *Lanata-Divaricata* showed the grouping of strains into clades of their respective type species (Supplementary Figures S3 and S4). In contrast, the *BenA* gene sequence survey for strain P05R2, previously identified as *T. koningiopsis*, returned limited data on type species, making phylogenetic tree construction unfeasible. However, partial sequences of the *CaM* gene, described as a secondary marker, have proven useful in the phylogenetic resolution of species in the genus *Trichoderma*, section *Trichoderma*, clade *Viride*, *Koningii* aggregate, as demonstrated by Samuels et al. [91]. In this context, constructing a phylogenetic tree with reference sequences for the *CaM* region allowed the alignment of P05R2 to the clade comprising *T. koningiopsis* strains (Supplementary Figure S5).

For bacteria, sequence similarity searches in the NCBI GenBank databases revealed the presence of cultivable members from various genera under the phyla *Proteobacteria* and *Firmicutes* (Table 3). *Burkholderia* was the most abundant genus among the isolates (41.67%), followed by *Bacillus* (25%). Regarding the distribution of strains at the collecting sites, both areas (P05 and P10) exhibited bacteria from the genera *Burkholderia* and *Bacillus*, sharing some common species such as *Burkholderia cepacia* and *Bacillus cereus*.

**Figure 1:**
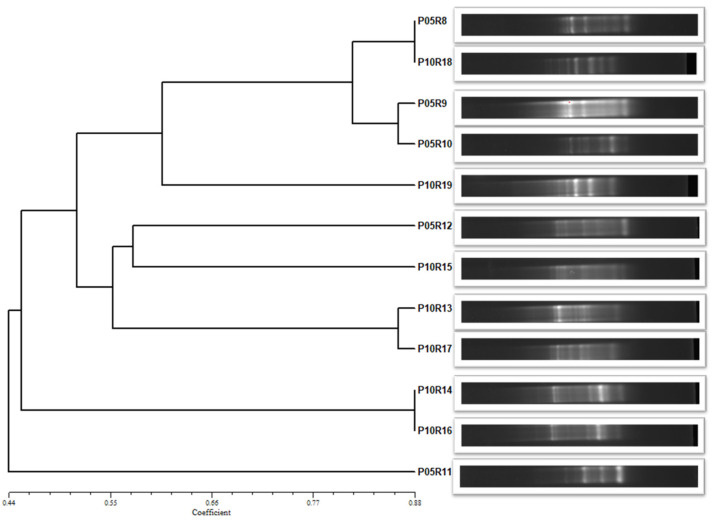
Dendrogram constructed from the BOX-PCR data profile of 12 bacterial isolates. The UPGMA method was used based on the similarity matrix. At 58% similarity, the isolates were grouped into 5 clusters.

In area P10, three distinct bacterial representatives were identified, including *Dyella japonica*, *Paraburkholderia* sp., and *Serratia marcescens*. Meanwhile, in area P05, unique representatives included *Bacillus* sp. and *Stenotrophomonas maltophilia*. The analysis of the dendrogram generated by the BOX-PCR technique (Figure 1) confirmed the existence of different bacterial groups, forming five clusters at 58% similarity. These results, combined with data from the NJ phylogenetic tree (Supplementary Figure S6) provided conclusive characteristics for distinguishing some of the formed groups. Among these, particular distinctions were noted in strain P10R19, identified as *Serratia marcescens*. This strain not only clustered with its type–species branch in the phylogenetic tree, but also exhibited unique characteristics in the BOX-PCR analysis, being allocated to a distinct taxon compared to the others. Another significant point was that strain P05R11, identified as *Stenotrophomonas maltophilia*, showed an exclusive profile in the BOX-PCR, being allocated to a distinct clade in the phylogenetic tree. Strains P05R8 and P10R18, identified both as *Bacillus cereus*, were grouped on the same branch in both the BOX dendrogram and the NJ tree, confirming the species correlation. While strains P05R9 (*Burkholderia cepacia*) and P05R10 (*Bacillus* sp.) presented similar profiles in the BOX-PCR, they were distinguishable via the NJ tree. Strains P05R12 and P10R15, identified as *Burkholderia* sp. and *Burkholderia cepacia*, shared the same branch in the BOX-PCR analysis, but were differentiated by their grouping into distinct taxa within the same clade in the phylogenetic tree. For strains P10R14 and P10R16, identified as *Paraburkholderia* sp. and *Burkholderia* sp., no significant differences were observed in the BOX-PCR profile. However, the phylogenetic interactions displayed by the NJ tree were conclusive in distinguishing the species.

### 3.4. Screening for Biosurfactants

**Figure 2:**
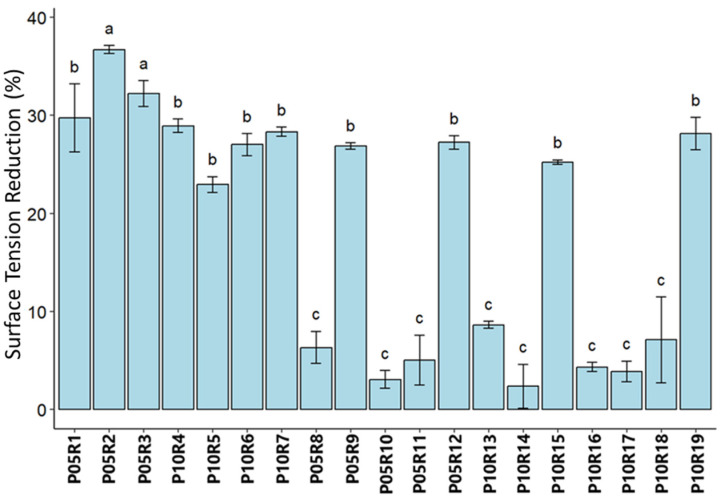
Results of screening for biosurfactant-producing isolates. Classification into groups according to Scott–Knott statistical analysis, where different letters indicate significant difference between the means of the treatments; *p*-value < 0.0001.

A total of 19 isolates, comprising 7 fungi and 12 bacteria, were screened for biosurfactant production in a minimal salt medium (MSM) using soybean oil as the sole carbon source. Surface tension reduction (STR) was the parameter used to select strains for subsequent stages, as an increase in STR is considered indicative of surfactant activity [40]. Based on our analysis, the average surface tension for the control medium was 54.57 mN·m^−1^. The supernatant of all fungal cultures demonstrated significant surfactant capabilities, evidenced by a STR > 20% (Figure 2).

Among the bacterial strains, only four showed relevant surfactant activity. For fungi, the strain identified as *T. koningiopsis* P05R2 stood out by reducing the medium’s surface tension by 36.6%. From the three best results, strains P05R1 and P05R3, identified as *P. janthinellum*, stood out, with an STR of 29.7% and 32.2%, respectively. Among the four bacterial representatives that demonstrated significant surfactant activity, strain *S. marcescens* P10R19 had the highest STR (28.1%). This was followed by strains of the genus *Burkholderia* (P05R9, P05R12, and P10R15), which reduced the medium’s surface tension by 26.8%, 27.2%, and 25.1%, respectively.

### 3.5. Screening for Laccases

**Figure 3:**
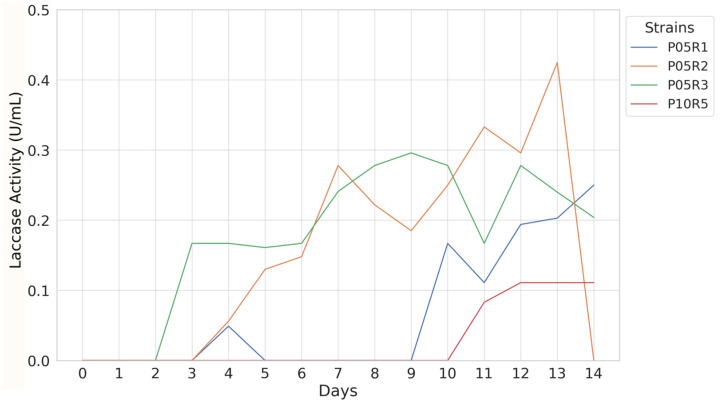
Monitoring extracellular laccase activity for fungal species.

Preliminary screening of all isolated strains revealed a greenish-blue color shift, characteristic of laccase presence, in four fungal (P05R1, P05R2, P05R3, and P10R5) and five bacterial strains (P05R8, P05R9, P05R11, P10R16, and P10R19). Only those isolates exhibiting a positive color change in this initial assay were selected for subsequent quantitative analysis of extracellular laccase activity. Among the fungal isolates selected, the quantitative assay detected the first sign of enzymatic activity after three days of culture, exhibited by *P. janthinellum* P05R3 (0.167 U/mL) (Figure 3).

On the fourth day of monitoring, other strains also showed evidence of enzymatic activity, including *P. janthinellum* P05R1 (0.049 U/mL) and *T. koningiopsis* P05R2 (0.056 U/mL). Overall, alternating peaks of activity were observed starting from the seventh day of cultivation. Among the fungi analyzed, *T. koningiopsis* P05R2 exhibited the highest enzymatic activity with a peak of 0.425 U/mL on the thirteenth day. During the monitoring period, two other peaks of enzymatic activity were observed prior to the maximum value for this strain: on the seventh day (0.278 U/mL) and on the eleventh day (0.333 U/mL). Additionally, significant signals were detected for *P. janthinellum* P05R3 on the ninth day, reaching 0.296 U/mL. The strains *P. janthinellum* P05R1 and *P.* sp. P10R5 showed later signs of enzymatic production, reaching maximum peaks on the fourteenth day, with 0.250 U/mL and 0.111 U/mL, respectively.

**Figure 4:**
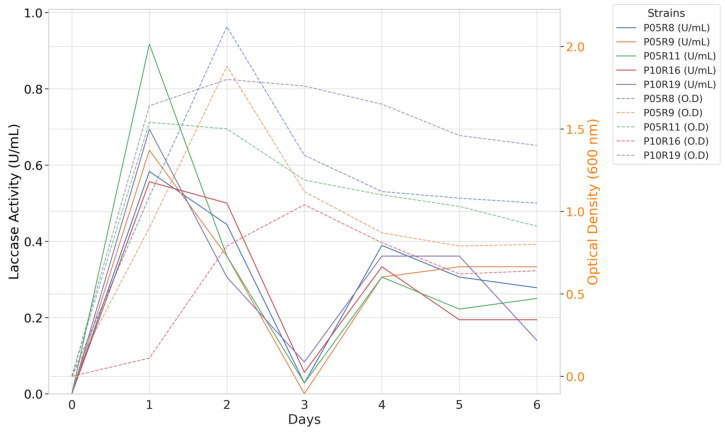
Monitoring laccase extracellular activity and microbial growth for bacterial species. Comparison of enzymatic activity relative to bacterial growth, measured by a spectrophotometer at 600 nm.

For bacteria, the enzymatic peak for all strains was reached within the first 24 h, as shown in Figure 4. Among the strains evaluated, *S. maltophilia* P05R11 stood out exhibiting a maximum enzymatic activity of 0.917 U/mL, followed by *S. marcescens* P10R19 with an activity of 0.694 U/mL. Other strains, including *B. cepacia* P05R9 (0.639 U/mL), *B. cereus* P05R8 (0.583 U/mL), and *Bacillus* sp. P10R16 (0.556 U/mL), also showed significant enzymatic activity. The optical density analysis of the cultures revealed the extracellular production of laccase throughout the growth phase, peaking during the exponential growth phase (12–24 h), followed by an abrupt decline in enzymatic activity as the cultures entered the stationary growth phase (48–72 h). Additional information regarding these findings is provided in Supplementary Tables S1 and S2.

### 3.6. Selection and Assembly of Microbial Consortia

The analysis of the growth dynamics among the species selected for this screening phase revealed microbial interactions resulting in moderate antagonistic effects (+), partially inhibiting colony development, as well as more intense antagonistic activities (+ +), which led to complete inhibition of culture growth (Table 4). Based on this, different combinations were proposed to formulate compatible microbial consortia by selecting species that demonstrated the ability to grow together in minimal medium supplemented with 1% *v*/*v* diesel and did not exhibit antagonistic interactions (−). Combined with the previous data on biosurfactant and laccase production, the following consortia were formulated: mix FFB1 (*Trichoderma koningiopsis* P05R2, *Penicillium janthinellum* P05R3, and *Serratia marcescens* P10R19), bacterial BB1 (*Stenotrophomonas maltophilia* P05R11 and *S. marcescens* P10R19), fungal FF1 (*T. koningiopsis* P05R2 and *P. janthinellum* P05R3), and mix FBB1 (*T. koningiopsis* P05R2, *S. marcescens* P10R19, and *Burkholderia cepacia* P05R9).

### 3.7. Biodegradation of Diesel Oil by Individual Strains and Microbial Consortia

**Figure 5:**
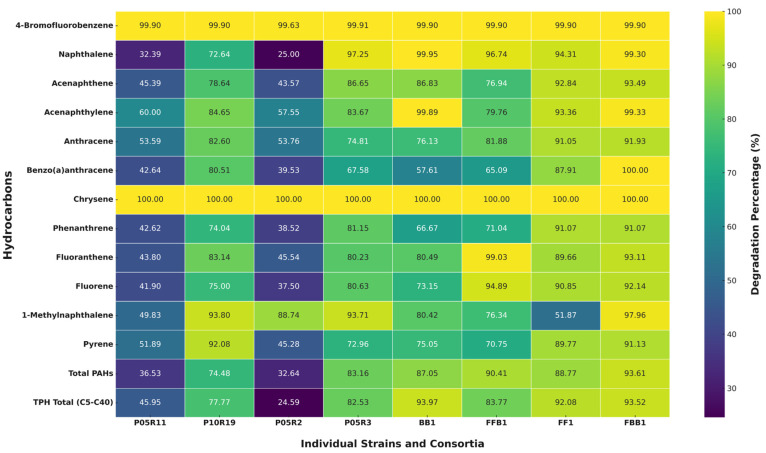
Efficiency of hydrocarbon degradation by individual strains and consortia. *p*-value < 0.05 according to ANOVA statistical analysis; mixed fungi-bacteria consortia FFB1 (*Trichoderma koningiopsis* P05R2, *Penicillium Janthinellum* P05R3, and *Serratia marcescens* P10R19), bacteria consortia BB1 (*Stenotrophomonas maltophilia* P05R11 and *S. marcescens* P10R19), fungi consortia FF1 (*T. koningiopsis* P05R2 and *P. Janthinellum* P05R3), mixed bacteria-fungi consortia FBB1 (*T. koningiopsis* P05R2, *S. marcescens* P10R19 and *Burkholderia cepacia* P05R9), individual fungal strain *T. koningiopsis* P05R2, individual fungal strain *P. Janthinellum* P05R3, individual bacterial strain *S. marcescens* P10R19, and individual bacterial strain *S. maltophilia* P05R11 are presented.

After 15 days of incubation in MSM, the degradation rates of total hydrocarbons and PAHs present in the remaining diesel oil differed greatly between treatments (Figure 5). An exception was had for 4-bromofluorobenzene and chrysene, which were 99.90% and 100% degraded by all strains and consortia combinations. Compared to the control samples, the individual bacterial strain *S. marcescens* P10R19 exhibited high removal rates for two–four-ring PAHs, including pyrene (92.08%), anthracene (82.60%), 1-methylnaphthalene (93.80%), fluoranthene (83.14%), and acenaphthylene (84.65%), demonstrating superior capability compared to other strains, such as *S. maltophilia* P05R11, which removed pyrene (51.89%), anthracene (53.59%), 1-methylnaphthalene (49.83%), fluoranthene (43.80%), and acenaphthylene (60%). Additionally, the *S. marcescens* P10R19 strain was able to degrade 77.77% of the TPH (C5–C40) and 74.48% of total PAHs, whereas *S. maltophilia* P05R11 degraded 45.95% of TPH (C5–C40) and 36.60% of total PAHs. When these two bacterial strains were combined in the BB1 consortium, significantly higher degradation percentages were observed, achieving 87.05% for total PAHs and 93.97% for TPH (C5–C40). The consortium showed improved removal rates for hydrocarbons such as naphthalene (99.95%), fluorene (73.15%), and phenanthrene (66.67%), surpassing the degradation capabilities of the individual strains.

Among the individually evaluated fungi, *P. janthinellum* P05R3 exhibited high degradation rates for total PAHs (83.16%) and TPH (C5–C40) at 82.53%, effectively removing hydrocarbons such as naphthalene (97.25%), acenaphthene (86.65%), and 1-methylnaphthalene (93.71%). In contrast, *T. koningiopsis* P05R2 achieved degradation rates greater than 60% only for specific hydrocarbons like 1-methylnaphthalene (88.74%). The combination of these two fungal strains in the FF1 consortium resulted in enhanced degradation rates, achieving 88.77% removal of total PAHs and 92.08% of TPH (C5–C40).

The mixed fungal–bacterial consortium FFB1, composed of *T. koningiopsis* P05R2, *P. janthinellum* P05R3, and *S. marcescens* P10R19, removed 90.41% of total PAHs and 83.77% of TPH (C5–C40). It achieved higher degradation rates for fluoranthene (99.03%) and fluorene (94.89%) compared to the BB1 and FF1 consortia. The consortium that exhibited the best results was FBB1, which included *T. koningiopsis* P05R2, *S. marcescens* P10R19, and *B. cepacia* P05R9 (the latter was not evaluated individually). According to the heatmap presented in Figure 5, this consortium achieved degradation rates of ≥91% for all analyzed PAHs, indicating a wide spectrum of degraded compounds with significantly higher rates.

Overall, Figure 5 shows that the different consortia tested achieved removal rates of ≥83% for total PAHs and TPH (C5–C40), indicating significant removal across a broad range of hydrocarbons. Furthermore, after the assay, we observed that aliquots of the cultures, serially diluted and plated, not only exhibited viability, but also showed no signs of antagonistic interactions between the strains within each consortium.

## 4. Discussion

A remarkable persistence of hydrocarbons was detected in area P05 after more than a decade since the original release. Concentrations above the limits established by the regulatory governmental agency were detected. These analyses reinforce the perspective that, although biodiesel is readily biodegradable, mixed compositions of diesel/biodiesel pose an imminent risk to the environment. In a study in the same study area, Ramos et al. [92] reported concentrations of benzene and benzo(a)pyrene above the regulatory values established after a period of 6.2 years since the experimental release of B20 diesel blends. According to the authors, these effects were attributed to the rapid consumption of electron acceptors, stimulated by the presence of biodiesel, forming an anaerobic degradation area, which delayed the removal of remaining mass. As pointed out by the literature, the metabolic pathways used by microorganisms during the transformation and degradation of hydrocarbon require a substantial amount of energy and predominantly occur under aerobic conditions [19,93]. In area P10, for instance, the bio-stimulation of native bacteria, through supplementation with iron oxides and ammonium acetate, provided significantly lower levels of benzene compared to the monitored natural attenuation of area P05 [51]. Indeed, our analysis found that benzene levels in area P10 were undetectable. However, the concentration of hydrocarbons such as xylenes, toluene, naphthalene, and anthracene remained above the standard levels.

Throughout the investigation, we found that among the identified fungal species, *Trichoderma koningiopsis* and *Penicillium pulvillorum* had not been previously described in areas impacted by petroleum hydrocarbons, making this the first report of these species in oil contaminated sites. The predominance of Ascomycetes in this initial analysis is consistent with findings from other studies that report the widespread distribution of genera like *Aspergillus*, *Penicillium*, and *Trichoderma* in hydrocarbon contaminated areas [27,94,95]. Characteristics such as tolerance to adverse environmental conditions, production of multi-enzyme complexes and surfactant molecules, exhibited by some species within these genera, contribute to their establishment in different habitats, promoting the transformation and degradation of a wide range of hydrocarbons [93,96,97].

In agreement with the present study, Covino et al. (2015) [98] reported that several Ascomycetes, including strains of *Fusarium*, *Penicillium,* and *Aspergillus*, were able to degrade approximately 79% of the aliphatic hydrocarbons present in contaminated soils. Another study of fungal communities in oil-contaminated sediments also demonstrated the role of species like *A. terreus*, *P. citreonigrum*, and *T. harzianum* in the degradation of aliphatic hydrocarbons [25]. Furthermore, an intervention in diesel-contaminated soil revealed that the high enzymatic activity, exhibited by fungal communities, was responsible for the degradation of 64% of four-ring PAHs, 28% of five-ring PAHs, 21% of two–three-ring PAHs and 16% of six-ring PAHs [99]. Although not reported for bioremediation, species such as *T. koningiopsis* are well-known for their use in the biocontrol of phytopathogens, including *Fusarium*, and in promoting plant growth through phosphate solubilization [100,101,102]. Therefore, the potential of the species in hydrocarbon degradation had not yet been explored.

With respect to bacteria, rapid development of strains was observed during the initial stages of in vitro sample enrichment, resulting in a greater number of isolates compared to fungi. In addition to the high concentration of hydrocarbon contaminants in the collecting areas, another factor that may have influenced the number of isolates includes the availability of oxygen and nutrients, which decreases with depth [103,104]. In this study, we found a prevalence of species from the genus *Burkholderia*, which was similarly documented by Ramos et al. (2014) [56] in experimental areas adjacent to P05 and P10. According to the findings of those authors, despite the predominance of *Burkholderia* in areas contaminated with B20 diesel, insignificant removal of BTEX and PAHs was detected during the period this bacterial species was dominant. It can be inferred that the proliferation of *Burkholderia* was associated with the consumption of acetate or methyl esters from the biodiesel. Somtrakoon et al. (2008) [105], on the other hand, associated *Burkholderia* (strain VUN10013) isolated from petrochemical-contaminated areas, with the removal of PAHs (fluoranthene and pyrene). In addition to these, more than 79 bacterial genera have been associated with the degradation of hydrocarbons, including *Achromobacter*, *Acinetobacter*, *Alkanindiges*, *Alteromonas*, *Enterobacter*, *Arthrobacter*, *Bacillus*, *Dietzia*, *Kocuria*, *Marinobacter*, *Mycobacterium*, *Pandoraea*, *Pseudomonas*, *Staphylococcus*, *Rhodococcus*, *Stenotrophomonas*, and *Serratia*, among others [6,106,107].

Among the species screened for biosurfactant production, *T. koningiopsis* P05R2 stood out, reducing the surface tension of the medium by 36.6%. This value was 3.3% higher than the threshold proposed by Walter, Syldatk, and Hausmann (2010) [108] for the selection of efficient biosurfactant-producing species, suggesting that *T. koningiopsis* P05R2 may be a promising species for this purpose. Although species from genera like *Trichoderma* and *Penicillium*, such as *T. citrinoviride*, *T. harzianum*, *T. atroviride*, *T. reesei*, *T. camerunense* [109,110,111], *P. islandicum*, *P. commune,* and *P. citrinum* [112,113,114], have been documented for biosurfactant production, there is an evident gap in the literature concerning studies that investigate the relationship between the production of biosurfactants by these genera and the enhanced hydrocarbon biodegradation. In a recent review, Rani et al. (2024) [115] also highlighted the scarcity of studies linking fungal biosurfactant production to hydrocarbon degradation and emphasized the need for more research in the field. While our study focused only on a preliminary screening for biosurfactant production, the results obtained, combined with the analysis of hydrocarbon degradation, provide novel insights into this underexplored area, offering additional information about these interactions and their potential.

Contrary to the case with fungi, the connection between biosurfactant-producing bacteria and enhanced hydrocarbon degradation has been documented by numerous authors [74,116,117,118]. A good example is *S. marcescens* P10R19, which had an STR of 28.1%. Using a different strain of the same species, Zhang et al. (2021) [119] demonstrated its capability of effectively degrading hydrocarbons while simultaneously producing biosurfactants. Other studies have also linked the emulsifying ability of *S. maltophilia* with enhanced degradation of PAHs [120]. In this regard, we identified *S. maltophilia* P05R11, a similar species that, despite its low STR of 5%, could be used for this purpose. It is also important to consider that, as this screening aimed at selecting candidates for bioremediation, optimal parameters for the production of these molecules were not evaluated. Therefore, the STR percentages achieved by the strains can likely be improved through further optimizations.

The monitoring of laccase activity revealed that all five bacterial strains evaluated reached peak enzyme production within the first 24 h, with significantly higher values compared to the fungal strains. This rapid production and higher activity are essential for efficient bioremediation, as it is well known that these enzymes catalyze the simultaneous oxidation of a large number of aromatic compounds and are directly responsible for degrading various PAHs [19,33]. However, most research for these applications focuses on laccases produced by fungi, citing the low expression and catalytic efficiency of bacterial laccases as justification [121,122]. Consequently, few studies cover the correlation between the production of these enzymes during bacterial growth and enhanced hydrocarbon degradation, including PAHs [122,123].

Recent research has explored the engineering of bacterial laccases through methods such as directed evolution and heterologous functional expression [96,124]. In this respect, genetically engineered strains of *E. coli*, such as BL21, have been used to overexpress laccase, achieving an enzyme activity of 9.5 U/mL. This laccase was utilized to enrich bacterial consortia composed of *Brevibacillus* sp. DL-1, *Bacillus* sp. DL-13 and *Acinetobacter schindleri* DL-34. The laccase-enriched consortia showed improved crude oil degradation efficiency, achieving 68.2% compared to 60.6% without the added laccase [125]. These results demonstrate superior enzymatic activity compared to *S. maltophilia* P05R11, the best-producing strain found in the present study, which achieved yields of 0.917 U/mL. Another approach more commonly found in the literature for the production of bacterial laccases is the optimization of cultivation parameters [80,126,127]. Following this, Galai, Limam and Marzouki (2009) [127,128] reported a peak enzymatic activity of 16 U/mL for the strain *S. maltophilia* AAP56 achieved after 24 h of cultivation. Using a similar methodology, Ali et al. (2022) [129] observed a maximum activity of 0.0733 U/mL for *Serratia proteamaculans* AORB19 after 48 h of cultivation. In the current investigation, the strain from the same genera, identified as *S. marcescens* P10R19, reached levels of 0.694 U/mL.

Among the analyzed fungi, we observed alternating peaks of enzymatic activity over a monitoring period of 14 days. *Trichoderma koningiopsis* P05R2 exhibited the highest peak (0.425 U/mL) on the thirteenth day of trial. The observations recorded were similar to those of Hölker, Dohse, and Höfer (2002) [130], who identified the first sign of enzymatic activity for a strain of *T. harzianum* on the fourth day of the culture, reaching maximum activity on the ninth day of cultivation (0.0138 U/mL). Similarly to the observations made for *T. koningiopsis* P05R2, a sharp decline in enzymatic activity started on the fourteenth day, rendering it undetectable after that.

In a similar study, a strain identified as *T. viride* isolated from soil samples contaminated with petroleum hydrocarbons, demonstrated the ability to produce extracellular laccases, reaching 23 U/mL. On the other hand, *Penicillium chrysogenum* showed an activity of 79 U/mL [131]. The production of these enzymes by fungi from the genera *Trichoderma* and *Penicillium*, along with their associated hydrocarbon degradation, can be found in various reports in the literature, demonstrating the potential of these genera for bioremediation [32,132,133].

Finally, the adoption of these screening methodologies enabled the formulation of distinct microbial consortia, which demonstrated the ability to efficiently remove a broad spectrum of PAHs and the TPH (C5–C40) present in diesel oil. The results of our analyses revealed a significant contrast between the capacity of the strains evaluated separately and their use in consortia, suggesting that the synergy between the strains was essential for the degradation of many hydrocarbon constituents. In agreement, numerous researchers highlight that the combined action of different microbial species, equipped with suitable metabolic mechanisms, can enhance the degradation efficiency and expand the range of compounds susceptible to microbial action [41,134,135].

In the present study, the use of microbial consortia demonstrated enhanced hydrocarbon degradation capabilities compared to individual strains. The combination of *S. maltophilia* P05R11 and *S. marcescens* P10R19 in the BB1 consortium resulted in significantly higher degradation percentages for both total PAHs and TPH (C5–C40). The benefit of using a bacterial consortium composed of the biosurfactant-producing strains *B. cereus* and *B. pumilus* over individual strains for the degradation of TPH, with special emphasis on PAHs, was also reported by Patowary et al. (2016) [44]. After 5 weeks of incubation, the authors observed the following degradation rates for listed hydrocarbons: naphthalene (70.87%), fluorene (61.79%), phenanthrene (58.98%), anthracene (100%), and TPH C5–C40 (84.15%). In this regard, the capacity of the BB1 consortium for hydrocarbon removal was significantly superior when comparing the degradation results obtained for hydrocarbons such as naphthalene (99.95%), fluorene (73.15%), phenanthrene (66.67%), and TPH C5–C40 (87.05%).

In a more recent study, Tripathi et al. (2023) [136] evaluated the degradation capabilities of individual bacterial strains of *Pseudomonas boreopolis* IITR108, *Microbacterium schleiferi* IITR109, *P. aeruginosa* IITR110 and *Bacillus velezensis* IITR111, which were isolated from crude oil. These strains showed 80–89% degradation of TPH (C8–C40) and 71–78% degradation of total PAHs. When these bacteria were combined into a consortium, they demonstrated degradation capabilities similar to those presented by the BB1 consortium, achieving 93.2% degradation of TPH (C8–C40) and 85.5% of total PAHs. Nnabuife et al. (2022) [137], on the other hand, reported that the degradation of TPH by the bacterial strains *P. aeruginosa* W15, *Providencia vermicola* W8, and *Serratia marcescens* W13 was higher when the strains were evaluated individually. These findings demonstrate that potential antagonistic effects may occur depending on the composition of the consortia, impacting the ability to remove particular compounds. In the present study, in addition to the results being improved by using the strains in consortia, it was also observed that the consortia remained viable and did not display any halos indicative of antagonistic interactions after the degradation assays.

In comparison to the BB1 bacterial consortium, the combination of *T. koningiopsis* P05R2 and *P. janthinellum* P05R3 in the FF1 consortium demonstrated enhanced degradation of total PAHs. Previous studies have highlighted the efficacy of fungal consortia in hydrocarbon degradation, emphasizing the role of ligninolytic enzymes. For example, Khan (2015) [138] reported that *P. janthinellum* was capable of degrading 96% of kerosene and 75% of diesel, with concomitant production of enzymes such as MnP and Lac, indicating that this species has a high potential for the application for bioremediation. Similarly, Vipotnik, Michelin, and Tavares (2021) [139] observed that a fungal consortium composed of the laccase-producing species *Aspergillus aegerita* and *P. chrysogenum* was capable of degrading 86% of fluorene when used as the sole carbon source. In comparison, the fungal consortium FF1 achieved a higher degradation rate for the same hydrocarbon, reaching removal rates of 90.85%.

Our findings further demonstrate that the integration of fungi and bacteria in mixed consortia can substantially enhance hydrocarbon degradation. These results are consistent with studies that have highlighted the cooperative roles of fungi and bacteria in mutualistic relationships, which facilitate hydrocarbon degradation. Ghorbannezhad, Moghimi, and Dastgheib (2021) [47] suggest that fungi play a primary role in the early stages of high-molecular-weight (HMW) PAHs and long-chain aliphatic degradation, while bacteria focus on removing the produced metabolites, completing the degradation process and thereby assisting fungi by reducing catabolic repression. The same authors also found that the addition of biosurfactants increased the degradation of pyrene and tetracosane by approximately 60.76% and 73.48%, respectively. Supporting the previous statement, Boochan et al. (2000) [140] investigated the biodegradation of HMW PAHs in liquid media using the bacteria *S. maltophilia* and the fungus *P. janthinellum*. They found that when evaluated individually, the bacteria utilized pyrene as their sole source of carbon and cometabolically mineralized benzo[a]pyrene when pyrene was present. Significant degradation and microbial growth on pyrene, chrysene, benz[a]anthracene, benzo[a]pyrene, and dibenz[a,h]anthracene occurred when the strains were co-cultivated, indicating that the mutual interaction between the strains was responsible for the enhanced degradation.

Similarly, *T. koningiopsis* P05R2, evaluated for the first time in this context, was not able to effectively degrade hydrocarbons when tested individually. However, when combined with different bacterial strains, the degradation of a broad spectrum of PAHs and TPH (C5–C40) was achieved. In this way, the cooperative role of *T. koningiopsis* in the degradation process highlights and reinforces the strain’s potential for future applications in microbial consortia designed for bioremediation. In another study, a consortium composed of five fungal isolates (*Aspergillus flavus* H6, *A. nomius* H7, *Rhizomucor variabilis* H9, *Trichoderma asperellum* H15, and *A. fumigatus* H19) and seven bacterial isolates (*Klebsiella pneumoniae* B1, *Enterobacter* sp. B3, *B. cereus* B4, *P. aeruginosa* B6, *Streptomyces* sp. B8, *Klebsiella* sp. B10, and *S. maltophilia* B14) was able to remove 87.76% of phenanthrene, 48.18% of pyrene, and 56.55% of benzo[a]pyrene after 14 days [141]. Thus, we can emphasize that the consortia developed in the present study covered a broader spectrum of degraded hydrocarbons compared to many other reports that deal with the application of mixed consortia for hydrocarbon remediation.

## 5. Conclusions

This study explored the potential of individual strains and novel mixed microbial consortia, composed of fungal and bacterial species isolated from areas contaminated with blends of diesel B20 (20% biodiesel and 80% diesel), in the degradation of PAHs and TPH (C5–C40) present in diesel oil. Among the biosurfactant and laccase-producing species selected for analysis, *Trichoderma koningiopsis* was identified for the first time as a candidate for bioremediation of petroleum hydrocarbons. The assays demonstrated that while some species showed promising individual performance, the combination of some strains in mixed consortia resulted in significantly greater degradation. A good example is the combination of *S. maltophilia* P05R11 and *S. marcescens* P10R19, reaching degradation rates of 87.05% for total PAHs and 93.97% for TPH (C5–C40), far superior when compared to the individual capacities of each strain alone. Similarly, the combination of the fungal strains *P. janthinellum* P05R3 and *T. koningiopsis* P05R2 resulted in 88.77% degradation of total PAHs and 92.08% of TPH (C5–C40). Nevertheless, the consortium that exhibited the best results was a mixed bacterial–fungal consortium, composed of *T. koningiopsis* P05R2, *S. marcescens* P10R19, and *B. cepacia* P05R9. This consortium was the only one to cover a degradation spectrum ≥91% for all PAHs analyzed, removing 93.61% of total PAHs and 93.52% of TPH (C5–C40). These results indicate that the combined action of different microbial species, equipped with different metabolic mechanisms, can significantly enhance the efficiency of hydrocarbon degradation. Therefore, our findings corroborate the premise that mixed microbial consortia are more effective in hydrocarbon remediation than isolated strains, covering a broader spectrum of degraded compounds. Future research should focus on optimizing these consortia and their application in contaminated soil microcosms to understand the extent of their degradation potential and behavior across different matrices.

## Figures and Tables

**Table 1:** Concentration of hydrocarbons at sampling sites.

Hydrocarbons (mg/g) *	Area [P05]	Area [P10]	Limit (CONAMA) **
Benzene	8.35 × 10^−5^	0	3.00 × 10^−5^
Toluene	5.17 × 10^−4^	1.92 × 10^−6^	1.40 × 10^−4^
Ethylbenzene	2.77 × 10^−6^	4.40 × 10^−4^	6.20 × 10^−6^
p-xylene	5.50 × 10^−6^	1.70 × 10^−6^	1.30 × 10^−4^
o-xylene	2.94 × 10^−6^	5.20 × 10^−4^	1.30 × 10^−4^
Naphthalene	7.49 × 10^−4^	6.02 × 10^−4^	1.20 × 10^−4^
Methyl-naphthalene	5.38 × 10^−6^	1.56 × 10^−2^	Ni
Acenaphthylene	3.85 × 10^−4^	5.59 × 10^−4^	Ni
Acenaphthene	8.21 × 10^−6^	7.75 × 10^−6^	Ni
Fluorene	2.97 × 10^−5^	2.78 × 10^−4^	Ni
Phenanthrene	5.67 × 10^−6^	1.44 × 10^−4^	3.30 × 10^−6^
Anthracene	1.91 × 10^−5^	3.90 × 10^−4^	3.90 × 10^−5^
Fluoranthene	6.64 × 10^−5^	3.98 × 10^−4^	Ni
Pyrene	6.96 × 10^−4^	7.66 × 10^−4^	Ni
Benzo(a)anthracene	6.89 × 10^−4^	3.28 × 10^−5^	2.50 × 10^−5^
Chrysene	5.59 × 10^−5^	2.95 × 10^−4^	8.10 × 10^−6^
Benzo(b)fluoranthene	0	1.07 × 10^−4^	Ni
Benzo(a)pyrene	2.31 × 10^−6^	3.92 × 10^−5^	8.70 × 10^−4^
Dibenzo(a,h)anthracene	1.37 × 10^−6^	1.79 × 10^−5^	8.0 × 10^−5^
Benzo(g,h,i)perylene	5.62 × 10^−6^	6.99 × 10^−6^	5.70 × 10^−4^
Indeno(1,2,3-CD)pyrene	1.21 × 10^−5^	3.34 × 10^−6^	3.10 × 10^−5^

* Gas chromatography results expressed in mg of hydrocarbon per gram of soil; ** Limit concentrations established by CONAMA; (Ni) not reported.

**Table 2:** Fungal species identified through sequencing of partial regions of the *β-tubulin* and *Calmodulin* genes.

Strain	Closest Match at NCBI (%)		Access number (GenBank)	
	*BenA*	*CaM*	*BenA*	*CaM*
P05R1	*Penicillium janthinellum* (99.80%)	*Penicillium janthinellum* (100%)	PP942382	PP942390
P05R2	*Trichoderma koningiopsis* (99.12%)	*Trichoderma koningiopsis* (95.88%)	PP942383	PP988131
P05R3	*Penicillium janthinellum* (98.07%)	*Penicillium janthinellum* (97.18%)	PP942384	PP942391
P10R4	*Penicillium pulvillorum* (99.19%)	*Penicillium pulvillorum* (98.72%)	PP942385	PP942392
P10R5	*Penicillium simplicissimum* (97.28%)	*Penicillium* sp. (96.82%)	PP942386	PP942393
P10R6	*Penicillium pulvillorum* (98.98%)	*Penicillium pulvillorum* (99.09%)	PP942387	PP942394
P10R7	*Penicillium pulvillorum* (99%)	*Penicillium pulvillorum* (98.57%)	PP942388	PP942395

**Table 3:** Bacterial species identified through sequencing of partial regions of the 16S rRNA gene.

Strain	Closest Match at NCBI (%)	Access Number (GenBank)
	*16S rRNA*	*16S rRNA*
P05R8	*Bacillus cereus* (97.19%)	PP952416
P05R9	*Burkholderia cepacia* (98.51%)	PP952605
P05R10	*Bacillus* sp. (99.33%)	PP952417
P05R11	*Stenotrophomonas maltophilia* (100%)	PP952606
P05R12	*Burkholderia* sp. (99.08%)	PP952607
P10R13	*Dyella japonica* (99.71%)	PP952608
P10R14	*Paraburkholderia* sp. (100%)	PP952609
P10R15	*Burkholderia cepacia* (100%)	PP952610
P10R16	*Burkholderia* sp. (100%)	PP952611
P10R17	*Burkholderia cepacia* (98.98%)	PP952612
P10R18	*Bacillus cereus* (99.22%)	PP952613
P10R19	*Serratia marcescens* (98.95%)	PP952418

**Table 4:** Assessment of antagonism activity between strains.

Bacteria vs. Fungus		Bacteria vs. Bacteria		Fungus vs. Fungus	
Strains	Inhibition	Strains	Inhibition	Strains	Inhibition
P05R1–P05R8	(−)	P05R9–P05R8	(−)	P05R1–P05R2	(−)
P05R1–P05R9	(+)	P05R9–P05R11	(+)	P05R1–P05R3	(+)
P05R1–P05R11	(+)	P05R9–P05R12	(+)	P05R1–P10R5	(+ +)
P05R1–P05R12	(−)	P05R9–P10R13	(−)	P05R1–P10R7	(+ +)
P05R1–P10R13	(−)	P05R8–P05R11	(+)	P05R2–P05R3	(−)
P05R1–P10R19	(−)	P05R8–P10R13	(−)	P05R2–P10R5	(+)
P05R2–P05R8	(−)	P05R11–P10R13	(−)	P05R2–P10R7	(+)
P05R2–P05R9	(−)	P05R12–P05R8	(+)	P05R3–P10R5	(−)
P05R2–P05R11	(+)	P05R12–P05R11	(−)	P05R3–P10R7	(−)
P05R2–P05R12	(+ +)	P05R12–P10R13	(−)	P10R5–P10R7	(−)
P05R2–P10R13	(−)	P10R19–P05R9	(−)		
P05R2–P10R19	(−)	P10R19–P05R12	(−)		
P05R3–P05R8	(+)	P10R19–P05R8	(−)		
P05R3–P05R9	(+)	P10R19–P05R11	(−)		
P05R3–P05R11	(+)	P10R19–P10R13	(−)		
P05R3–P05R12	(+)				
P05R3–P10R13	(−)				
P05R3–P10R19	(−)				
P10R5–P05R8	(+)				
P10R5–P05R9	(+ +)				
P10R5–P05R11	(−)				
P10R5–P05R12	(+ +)				
P10R5–P10R13	(−)				
P10R5–P10R19	(−)				
P10R7–P05R8	(−)				
P10R7–P05R9	(+ +)				
P10R7–P05R11	(−)				
P10R7–P05R12	(+)				
P10R7–P10R13	(+)				
P10R7–P05R16	(−)				
P10R7–P10R19	(−)				

The absence of inhibition zones or antagonistic effects between co-cultures is represented by (−); moderate antagonistic effects are represented by (+); strong antagonistic effects resulting in complete inhibition are represented by (+ +).

## Data Availability

Data are contained within the article.

## Supplementary Materials

The following supporting information can be downloaded at https://www.mdpi.com/article/10.3390/toxics12120913/s1: Table S1: Bacterial laccase activity over time; Supplementary Table S2: Fungal laccase activity over time; Supplementary Figure S1: Surface tension measurement using the pendant drop assay on a goniometer; Figure S2: Macroscopic and microscopic morphological characterization of fungal isolates on Malt Extract Agar (MEA), Czapek Yeast Extract Agar (CYA), and Yeast Extract Sucrose Agar (YES); Figure S3: Phylogenetic tree constructed based on the partial β-tubulin (*BenA*) gene sequence data for the Lanata-Divaricata section of the genus *Penicillium*. The Kimura model (K2 + G) was used, employing distance data by the Maximum-Likelihood (ML) method and bootstrap analysis with 1.000 replicates, using *Penicillium glabrum* as the outgroup; Figure S4: Phylogenetic tree constructed based on partial Calmodulin (*CaM*) gene sequence data for the Lanata-Divaricata section of the genus *Penicillium*. The Kimura model (K2 + G) was used, employing distance data by the Maximum-Likelihood (ML) method and bootstrap analysis with 1.000 replicates, using *Penicillium glabrum* as the outgroup; Figure S5: Phylogenetic tree constructed based on partial Calmodulin (*CaM*) gene sequence data for the section *Trichoderma* clade *viride*, *Koningii* aggregate. The Kimura model (K2 + G) was used, employing distance data by the Maximum-Likelihood (ML) method and bootstrap analysis with 1.000 replicates, using *Trichoderma hamatum* as the outgroup; Figure S6: Phylogenetic tree constructed based on partial 16S rRNA gene sequence data for the type species of the referred lineages. The Kimura model (K2 + G) was used, employing distance data by the Neighbor-Joining (NJ) method and bootstrap analysis with 1.000 replicates, using *Deinococcus radiodurans* as the outgroup.

## References

[1] Ladle R.J., Malhado A.C.M., Campos-Silva J.V., Pinheiro B.R. (2020). Brazil’s mystery oil spill: An ongoing social disaster. Nature.

[2] Zhang B., Matchinski E.J., Chen B., Ye X., Jing L., Lee K. (2018). Marine oil spills-oil pollution, sources and effects. World Seas: An Environmental Evaluation Volume III: Ecological Issues and Environmental Impacts.

[3] Carmo E.H., Teixeira M.G. (2020). Technological disasters and public health emergencies: The case of oil spill on the Brazilian coast. Cad. Saude Publica.

[4] Schobert H. (2013). Composition, classification, and properties of petroleum. Chemistry of Fossil Fuels and Biofuels.

[5] Barathi S., Gitanjali J., Rathinasamy G., Sabapathi N., Aruljothi K.N., Lee J., Kandasamy S. (2023). Recent trends in polycyclic aromatic hydrocarbons pollution distribution and counteracting bio-remediation strategies. Chemosphere.

[6] Patel A.B., Shaikh S., Jain K.R., Desai C., Madamwar D. (2020). Polycyclic Aromatic Hydrocarbons: Sources, Toxicity, and Remediation Approaches. Front. Microbiol..

[7] Delgado-Saborit J.M., Stark C., Harrison R.M. (2011). Carcinogenic potential, levels and sources of polycyclic aromatic hydrocarbon mixtures in indoor and outdoor environments and their implications for air quality standards. Environ. Int..

[8] Mastrangelo G., Fadda E., Marzia V. (1996). Polycyclic aromatic hydrocarbons and cancer in man. Environ. Health Perspect..

[9] Mulla S.I., Bagewadi Z.K., Faniband B., Bilal M., Chae J.C., Bankole P.O., Saratale G.D., Bhargava R.N., Gurumurthy D.M. (2023). Various strategies applied for the removal of emerging micropollutant sulfamethazine: A systematic review. Environ. Sci. Pollut. Res..

[10] Dzionek A., Wojcieszyńska D., Guzik U. (2016). Natural carriers in bioremediation: A review. Electron. J. Biotechnol..

[11] Srivastava R.K., Shetti N.P., Reddy K.R., Aminabhavi T.M. (2020). Biofuels, biodiesel and biohydrogen production using bioprocesses. A review. Environ. Chem. Lett..

[12] Chauhan S.K., Gangopadhyay S., Singh N. (2009). Environmental aspects of biofuels in road transportation. Environ. Chem. Lett..

[13] Council Anticipates Higher Biodiesel Percentage in Diesel and Encourages Energy Transition, 2024. https://www.gov.br/mdic/pt-br/assuntos/noticias/2023/dezembro/cnpe-aprova-antecipacao-do-b14-para-marco-de-2024-e-b15-para-marco-de-2025-incentivando-a-producao-de-biocombustiveis-e-a-transicao-energetica.

[14] Kohlhepp G. (2010). Análise da situação da produção de etanol e biodiesel no Brasil. Estud. Avançados.

[15] Khan N., Warith M.A., Luk G. (2007). A Comparison of acute toxicity of biodiesel, biodiesel blends, and diesel on aquatic organisms. J. Air Waste Manag. Assoc..

[16] Demirbaş A. (2009). Biodegradability of biodiesel and petrodiesel fuels. Energy Sources Part A Recover. Util. Environ. Eff..

[17] Zungum I.U., Imam T.S. (2021). Ecotoxicity and Associated Threat of Polycyclic Aromatic Hydrocarbons (PAHs) to Biodiversity: A Review. Preprints.

[18] Mishra P., Kiran N.S., Romanholo Ferreira L.F., Yadav K.K., Mulla S.I. (2023). New insights into the bioremediation of petroleum contaminants: A systematic review. Chemosphere.

[19] Thacharodi A., Hassan S., Singh T., Mandal R., Chinnadurai J., Khan H.A., Hussain M.A., Brindhadevi K., Pugazhendhi A. (2023). Bioremediation of polycyclic aromatic hydrocarbons: An updated microbiological review. Chemosphere.

[20] Fulekar M.H. (2017). Microbial degradation of petrochemical waste-polycyclic aromatic hydrocarbons. Bioresour. Bioprocess.

[21] Gan J.S., Bilal M., Li X.B., Hussain Shah S.Z., Mohamed B.A., Hadibarata T., Cheng H. (2022). Peroxidases-based enticing biotechnological platforms for biodegradation and biotransformation of emerging contaminants. Chemosphere.

[22] Fritsche W., Hofrichter M. (2008). Aerobic Degradation by Microorganisms. Biotechnology: Second, Completely Revised Edition.

[23] Wu M., Li W., Dick W.A., Ye X., Chen K., Kost D., Chen L. (2017). Bioremediation of hydrocarbon degradation in a petroleum-contaminated soil and microbial population and activity determination. Chemosphere.

[24] Suja F., Rahim F., Taha M.R., Hambali N., Rizal Razali M., Khalid A., Hamzah A. (2014). Effects of local microbial bioaugmentation and biostimulation on the bioremediation of total petroleum hydrocarbons (TPH) in crude oil contaminated soil based on laboratory and field observations. Int. Biodeterior. Biodegrad..

[25] Bovio E., Gnavi G., Prigione V., Spina F., Denaro R., Yakimov M., Calogero R., Crisafi F., Varese G.C. (2017). The culturable mycobiota of a Mediterranean marine site after an oil spill: Isolation, identification and potential application in bioremediation. Sci. Total Environ..

[26] Benguenab A., Chibani A. (2020). Biodegradation of petroleum hydrocarbons by filamentous fungi (*Aspergillus ustus* and *Purpureocillium lilacinum*) isolated from used engine oil contaminated soil. Acta Ecol. Sin..

[27] Medaura M.C., Guivernau M., Moreno-Ventas X., Prenafeta-Boldú F.X., Viñas M. (2021). Bioaugmentation of Native Fungi, an Efficient Strategy for the Bioremediation of an Aged Industrially Polluted Soil with Heavy Hydrocarbons. Front. Microbiol..

[28] Barnes N.M., Khodse V.B., Lotlikar N.P., Meena R.M., Damare S.R. (2018). Bioremediation potential of hydrocarbon-utilizing fungi from select marine niches of India. 3 Biotech.

[29] Yuniati M.D. (2018). Bioremediation of petroleum-contaminated soil: A Review. IOP Conference Series: Earth and Environmental Science.

[30] Akhtar N., Mannan M.A. (2020). ul Mycoremediation: Expunging environmental pollutants. Biotechnol. Rep..

[31] Siles J.A., Margesin R. (2018). Insights into microbial communities mediating the bioremediation of hydrocarbon-contaminated soil from an Alpine former military site. Appl. Microbiol. Biotechnol..

[32] Kadri T., Rouissi T., Kaur Brar S., Cledon M., Sarma S., Verma M. (2017). Biodegradation of polycyclic aromatic hydrocarbons (PAHs) by fungal enzymes: A review. J. Environ. Sci..

[33] Giardina P., Faraco V., Pezzella C., Piscitelli A., Vanhulle S., Sannia G. (2010). Laccases: A never-ending story. Cell. Mol. Life Sci..

[34] Aranda E., Godoy P., Reina R., Badia-Fabregat M., Rosell M., Marco-Urrea E., García-Romera I. (2017). Isolation of Ascomycota fungi with capability to transform PAHs: Insights into the biodegradation mechanisms of Penicillium oxalicum. Int. Biodeterior. Biodegradation.

[35] Pozdnyakova N., Dubrovskaya E., Chernyshova M., Makarov O., Golubev S., Balandina S., Turkovskaya O. (2018). The degradation of three-ringed polycyclic aromatic hydrocarbons by wood-inhabiting fungus Pleurotus ostreatus and soil-inhabiting fungus Agaricus bisporus. Fungal Biol..

[36] Ghosal D., Ghosh S., Dutta T.K., Ahn Y. (2016). Current state of knowledge in microbial degradation of polycyclic aromatic hydrocarbons (PAHs): A review. Front. Microbiol..

[37] Parthipan P., Elumalai P., Sathishkumar K., Sabarinathan D., Murugan K., Benelli G., Rajasekar A. (2017). Biosurfactant and enzyme mediated crude oil degradation by Pseudomonas stutzeri NA3 and Acinetobacter baumannii MN3. 3 Biotech.

[38] Hickey W.J., Chen S., Zhao J. (2012). The phn island: A new genomic island encoding catabolism of polynuclear aromatic hydrocarbons. Front. Microbiol..

[39] Imam A., Kumar Suman S., Kanaujia P.K., Ray A. (2022). Biological machinery for polycyclic aromatic hydrocarbons degradation: A review. Bioresour. Technol..

[40] da Silva A.F., Banat I.M., Giachini A.J., Robl D. (2021). Fungal biosurfactants, from nature to biotechnological product: Bioprospection, production and potential applications. Bioprocess Biosyst. Eng..

[41] Ławniczak Ł., Woźniak-Karczewska M., Loibner A.P., Heipieper H.J., Chrzanowski Ł. (2020). Microbial degradation of hydrocarbons—Basic principles for bioremediation: A review. Molecules.

[42] Zhou M.F., Yuan X.Z., Zhong H., Liu Z.F., Li H., Jiang L.L., Zeng G.M. (2011). Effect of biosurfactants on laccase production and phenol biodegradation in solid-state fermentation. Appl. Biochem. Biotechnol..

[43] Mnif I., Ellouze-Chaabouni S., Ayedi Y., Ghribi D. (2014). Treatment of Diesel- and Kerosene-Contaminated Water by *B. subtilis* SPB1 Biosurfactant-Producing Strain. Water Environ. Res..

[44] Patowary K., Patowary R., Kalita M.C., Deka S. (2016). Development of an efficient bacterial consortium for the potential remediation of hydrocarbons from contaminated sites. Front. Microbiol..

[45] Kamyabi A., Nouri H., Moghimi H. (2017). Synergistic Effect of *Sarocladium* sp. and *Cryptococcus* sp. Co-Culture on Crude Oil Biodegradation and Biosurfactant Production. Appl. Biochem. Biotechnol..

[46] Ghorbannezhad H., Moghimi H., Dastgheib S.M.M. (2018). Evaluation of heavy petroleum degradation using bacterial-fungal mixed cultures. Ecotoxicol. Environ. Saf..

[47] Ghorbannezhad H., Moghimi H., Dastgheib S.M.M. (2021). Evaluation of pyrene and tetracosane degradation by mixed-cultures of fungi and bacteria. J. Hazard. Mater..

[48] El Hanafy A.A.E.M., Anwar Y., Mohamed S.A., Al-Garni S.M.S., Sabir J.S.M., Abuzinadah O.A., Al Mehdar H., Alfaidi A.W., Ahmed M.M.M. (2016). Isolation and identification of bacterial consortia responsible for degrading oil spills from the coastal area of Yanbu, Saudi Arabia. Biotechnol. Biotechnol. Equip..

[49] Atakpa E.O., Zhou H., Jiang L., Ma Y., Liang Y., Li Y., Zhang D., Zhang C. (2022). Improved degradation of petroleum hydrocarbons by co-culture of fungi and biosurfactant-producing bacteria. Chemosphere.

[50] Ameen F., Moslem M., Hadi S., Al-Sabri A.E. (2016). Biodegradation of diesel fuel hydrocarbons by mangrove fungi from Red Sea Coast of Saudi Arabia. Saudi J. Biol. Sci..

[51] Müller J.B., Ramos D.T., Larose C., Fernandes M., Lazzarin H.S.C., Vogel T.M., Corseuil H.X. (2017). Combined iron and sulfate reduction biostimulation as a novel approach to enhance BTEX and PAH source-zone biodegradation in biodiesel blend-contaminated groundwater. J. Hazard. Mater..

[52] USEPA, U.S.E.P.A (2007). SW-846 Test Method 3550C: Ultrasonic Extraction, SW-846 Compendium. https://www.epa.gov/hw-sw846/sw-846-test-method-3550c-ultrasonic-extraction.

[53] USEPA (2003). Volatile Organic Compounds in Various Sample Matrices Using Equilibrium Headspace Analysis.

[54] (1996). USEPA SW-846 Test Method 8000D: Determinative Chromatographic Separations|Hazardous Waste Test Methods/SW-846|US EPA. U.S. Environmental Protection Agency. https://www.epa.gov/hw-sw846/sw-846-test-method-8000d-determinative-chromatographic-separations.

[55] Corseuil H.X., Monier A.L., Fernandes M., Schneider M.R., Nunes C.C., Do Rosario M., Alvarez P.J.J. (2011). BTEX plume dynamics following an ethanol blend release: Geochemical footprint and thermodynamic constraints on natural attenuation. Environ. Sci. Technol..

[56] Ramos D.T., da Silva M.L.B., Nossa C.W., Alvarez P.J.J., Corseuil H.X. (2014). Assessment of microbial communities associated with fermentative-methanogenic biodegradation of aromatic hydrocarbons in groundwater contaminated with a biodiesel blend (B20). Biodegradation.

[57] Poddar K., Sarkar D., Sarkar A. (2019). Construction of potential bacterial consortia for efficient hydrocarbon degradation. Int. Biodeterior. Biodegrad..

[58] Bushnell L.D., Haas H.F. (1941). The Utilization of Certain Hydrocarbons by Microorganisms. J. Bacteriol..

[59] Adams E. (1975). Studies in gram staining. Biotech. Histochem..

[60] García de Lomas J., Marín F., Altuna A., Cuadrado E. (1981). Méthode simple et rapide de réalisation de microculture en identification mycologique. Mycopathologia.

[61] Pitt J.I. (1979). The Genus Penicillium and its Teleomorphic States Eupenicillium and Talaromyces. 634 S., 132 Abb. London-New York-Toronto-Sydney-San Francisco 1979. Academic Press. $92.00. J. Basic Microbiol..

[62] Frisvad J.C. (1981). Physiological criteria and mycotoxin production as aids in identification of common asymmetric penicillia. Appl. Environ. Microbiol..

[63] Glass N.L., Donaldson G.C. (1995). Development of primer sets designed for use with the PCR to amplify conserved genes from filamentous ascomycetes. Appl. Environ. Microbiol..

[64] Hong S.B., Cho H.S., Shin H.D., Frisvad J.C., Samson R.A. (2006). Novel Neosartorya species isolated from soil in Korea. Int. J. Syst. Evol. Microbiol..

[65] Visagie C.M., Houbraken J., Frisvad J.C., Hong S.B., Klaassen C.H.W., Perrone G., Seifert K.A., Varga J., Yaguchi T., Samson R.A. (2014). Identification and nomenclature of the genus Penicillium. Stud. Mycol..

[66] Lane D.J. (1991). 16S/23S rRNA Sequencing. Nucleic acid Tech. Bact. Syst..

[67] Turner S., Pryer K.M., Miao V.P.W., Palmer J.D. (1999). Investigating deep phylogenetic relationships among cyanobacteria and plastids by small subunit rRNA sequence analysis. J. Eukaryot. Microbiol..

[68] Kumar S., Stecher G., Tamura K. (2016). MEGA7: Molecular evolutionary genetics analysis version 7.0. molecular biology and evolution. Mol. Biol. Evol..

[69] Saitou N., Nei M. (1987). The neighbor-joining method: A new method for reconstructing phylogenetic trees. Mol. Biol. Evol..

[70] Felsenstein J. (1981). Evolutionary trees from DNA sequences: A maximum likelihood approach. J. Mol. Evol..

[71] Versalovic J. (1994). Genomic fingerprinting of bacteria using repetitive sequence-based polymerase chain reaction. Methods Mol. Cell. Biol..

[72] Sokal R.R. (1958). A statistical method for evaluating systematic relationships. Univ. Kans. Sci. Bull..

[73] Rohlf F.J. (2000). NTSYpc Numerical Taxonomy and Multivariate Analysis System Version 2.1 User Guide.

[74] Ray M., Kumar V., Banerjee C., Gupta P., Singh S., Singh A. (2021). Investigation of biosurfactants produced by three indigenous bacterial strains, their growth kinetics and their anthracene and fluorene tolerance. Ecotoxicol. Environ. Saf..

[75] Othman A.R., Ismail N.S., Abdullah S.R.S., Hasan H.A., Kurniawan S.B., Sharuddin S.S.N., Ismail N. (2022). Potential of indigenous biosurfactant-producing fungi from real crude oil sludge in total petroleum hydrocarbon degradation and its future research prospects. J. Environ. Chem. Eng..

[76] Lecomte Du Noüy P. (1925). An interfacial tensiometer for universal use. J. Gen. Physiol..

[77] Ebnesajjad S. (2010). Surface Tension and Its Measurement. Handbook of Adhesives and Surface Preparation: Technology, Applications and Manufacturing.

[78] Chandankere R., Yao J., Cai M., Masakorala K., Jain A.K., Choi M.M.F. (2014). Properties and characterization of biosurfactant in crude oil biodegradation by bacterium *Bacillus methylotrophicus* USTBa. Fuel.

[79] Karp S.G., Faraco V., Amore A., Birolo L., Giangrande C., Soccol V.T., Pandey A., Soccol C.R. (2012). Characterization of laccase isoforms produced by *Pleurotus ostreatus* in solid state fermentation of sugarcane bagasse. Bioresour. Technol..

[80] Rajeswari M., Vennila K., Bhuvaneswari V. (2015). Optimization of laccase production media by *Bacilllus cereus* TSS1 using Box-Behnken design. Int. J. Chem. Pharm. Sci..

[81] Hou H., Zhou J., Wang J., Du C., Yan B. (2004). Enhancement of laccase production by Pleurotus ostreatus and its use for the decolorization of anthraquinone dye. Process Biochem..

[82] Johannes C., Majcherczyk A. (2000). Laccase activity tests and laccase inhibitors. J. Biotechnol..

[83] Leonowicz A., Grzywnowicz K. (1981). Quantitative estimation of laccase forms in some white-rot fungi using syringaldazine as a substrate. Enzyme Microb. Technol..

[84] Wunder T., Kremer S., Sterner O., Anke H. (1994). Metabolism of the polycyclic aromatic hydrocarbon pyrene by *Aspergillus niger* SK 9317. Appl. Microbiol. Biotechnol..

[85] Zafra G., Absalón Á.E., Anducho-Reyes M.Á., Fernandez F.J., Cortés-Espinosa D.V. (2017). Construction of PAH-degrading mixed microbial consortia by induced selection in soil. Chemosphere.

[86] Ganesh A., Lin J. (2009). Diesel degradation and biosurfactant production by Gram-positive isolates. African J. Biotechnol..

[87] Ebadi A., Ghavidel A., Khoshkholgh Sima N.A., Heydari G., Ghaffari M.R. (2021). New strategy to increase oil biodegradation efficiency by selecting isolates with diverse functionality and no antagonistic interactions for bacterial consortia. J. Environ. Chem. Eng..

[88] Eicheelberger J.W., Behymer T.D., Budde W.L. (1995). METHOD 525.2 Deterimination of Organic Compounds in Drinking Water by Liquid-Solid Extraction and Capillary Column Gas Chromatography/Mass Spectomery.

[89] Jelihovschi E., Faria J.C., Allaman I.B. (2014). ScottKnott: A Package for Performing the Scott-Knott Clustering Algorithm in R. Trends Comput. Appl. Math..

[90] R Core Team (2021). R: A Language and Environment for Statistical Computing.

[91] Samuels G.J., Dodd S., Lu B.S., Petrini O., Schroers H.J., Druzhinina I.S. (2006). The Trichoderma koningii aggregate species. Stud. Mycol..

[92] Ramos D.T., Lazzarin H.S.C., Alvarez P.J.J., Vogel T.M., Fernandes M., do Rosário M., Corseuil H.X. (2016). Biodiesel presence in the source zone hinders aromatic hydrocarbons attenuation in a B20-contaminated groundwater. J. Contam. Hydrol..

[93] Pandey P., Pathak H., Dave S. (2016). Microbial ecology of hydrocarbon degradation in the soil: A review. Res. J. Environ. Toxicol..

[94] Ferreira J.A., Varjani S., Taherzadeh M.J. (2020). A Critical Review on the Ubiquitous Role of Filamentous Fungi in Pollution Mitigation. Curr. Pollut. Reports.

[95] Horel A., Schiewer S. (2020). Microbial degradation of different hydrocarbon fuels with mycoremediation of volatiles. Microorganisms.

[96] Narayanan M., Ali S.S., El-Sheekh M. (2023). A comprehensive review on the potential of microbial enzymes in multipollutant bioremediation: Mechanisms, challenges, and future prospects. J. Environ. Manag..

[97] da Silva A.F., Banat I.M., Robl D., Giachini A.J. (2023). Fungal bioproducts for petroleum hydrocarbons and toxic metals remediation: Recent advances and emerging technologies. Bioprocess Biosyst. Eng..

[98] Covino S., D’Annibale A., Stazi S.R., Cajthaml T., Čvančarová M., Stella T., Petruccioli M. (2015). Assessment of degradation potential of aliphatic hydrocarbons by autochthonous filamentous fungi from a historically polluted clay soil. Sci. Total Environ..

[99] Borowik A., Wyszkowska J., Oszust K. (2017). Functional diversity of fungal communities in soil contaminated with diesel oil. Front. Microbiol..

[100] Tandon A., Fatima T., Anshu, Shukla D., Tripathi P., Srivastava S., Singh P.C. (2020). Phosphate solubilization by Trichoderma koningiopsis (NBRI-PR5) under abiotic stress conditions. J. King Saud Univ. Sci..

[101] Yu C., Luo X. (2020). Trichoderma koningiopsis controls Fusarium oxysporum causing damping-off in Pinus massoniana seedlings by regulating active oxygen metabolism, osmotic potential, and the rhizosphere microbiome. Biol. Control.

[102] Ulrich A., Lerin L.A., Camargo A.F., Scapini T., Diering N.L., Bonafin F., Gasparetto I.G., Confortin T.C., Sansonovicz P.F., Fabian R.L. (2021). Alternative bioherbicide based on Trichoderma koningiopsis: Enzymatic characterization and its effect on cucumber plants and soil organism. Biocatal. Agric. Biotechnol..

[103] Aydin S., Karaçay H.A., Shahi A., Gökçe S., Ince B., Ince O. (2017). Aerobic and anaerobic fungal metabolism and Omics insights for increasing polycyclic aromatic hydrocarbons biodegradation. Fungal Biol. Rev..

[104] Al-Turki A.I. (2009). Microbial polycyclic aromatic hydrocarbons degradation in soil. Res. J. Environ. Toxicol..

[105] Somtrakoon K., Suanjit S., Pokethitiyook P., Kruatrachue M., Lee H., Upatham S. (2008). Phenanthrene stimulates the degradation of pyrene and fluoranthene by Burkholderia sp. VUN10013. World J. Microbiol. Biotechnol..

[106] Sierra-Garcia I.N., Oliveira V.M., Sierra-Garcia I.N., de Oliveira V.M. (2013). Microbial Hydrocarbon Degradation: Efforts to Understand Biodegradation in Petroleum Reservoirs. Biodegradation–Engineering and Technology.

[107] Imron M.F., Kurniawan S.B., Ismail N.I., Abdullah S.R.S. (2020). Future challenges in diesel biodegradation by bacteria isolates: A review. J. Clean. Prod..

[108] Walter V., Syldatk C., Hausmann R. (2010). Screening concepts for the isolation of biosurfactant producing microorganisms. Adv. Exp. Med. Biol..

[109] Piegza M., Pietrzykowska J., Trojan-Piegza J., Łaba W. (2021). Biosurfactants from trichoderma filamentous fungi—A preliminary study. Biomolecules.

[110] Martinho V., dos Santos Lima L.M., Barros C.A., Ferrari V.B., Passarini M.R.Z., Santos L.A., de Souza Sebastianes F.L., Lacava P.T., de Vasconcellos S.P. (2019). Enzymatic potential and biosurfactant production by endophytic fungi from mangrove forest in Southeastern Brazil. AMB Express.

[111] Pitocchi R., Cicatiello P., Birolo L., Piscitelli A., Bovio E., Cristina Varese G., Giardina P. (2020). Cerato-Platanins from Marine Fungi as Effective Protein Biosurfactants and Bioemulsifiers. Int. J. Mol. Sci..

[112] Kumar V., Kumar H., Vishal V., Lal S. (2023). Studies on the morphology, phylogeny, and bioremediation potential of *Penicillium citrinum* and *Paecilomyces variotii* (Eurotiales) from oil-contaminated areas. Arch. Microbiol..

[113] Al-Hawash A.B., Zhang X., Ma F. (2019). Removal and biodegradation of different petroleum hydrocarbons using the filamentous fungus *Aspergillus* sp. RFC-1. Microbiologyopen.

[114] Kulkarni S.S., Nene S.N., Joshi K.S. (2020). Exploring malted barley waste for fungi producing surface active proteins like hydrophobins. SN Appl. Sci..

[115] Rani M.H.S., Nandana R.K., Khatun A., Brindha V., Midhun D., Gowtham P., Mani S.S.D., Kumar S.R., Aswini A., Muthukumar S. (2024). Three strategy rules of filamentous fungi in hydrocarbon remediation: An overview. Biodegradation.

[116] Bezza F.A., Chirwa E.M.N. (2017). Pyrene biodegradation enhancement potential of lipopeptide biosurfactant produced by Paenibacillus dendritiformis CN5 strain. J. Hazard. Mater..

[117] Gupta B., Puri S., Thakur I.S., Kaur J. (2020). Enhanced pyrene degradation by a biosurfactant producing Acinetobacter baumannii BJ5: Growth kinetics, toxicity and substrate inhibition studies. Environ. Technol. Innov..

[118] Rehman R., Ali M.I., Ali N., Badshah M., Iqbal M., Jamal A., Huang Z. (2021). Crude oil biodegradation potential of biosurfactant-producing *Pseudomonas aeruginosa* and *Meyerozyma* sp.. J. Hazard. Mater..

[119] Zhang K., Tao W., Lin J., Wang W., Li S. (2021). Production of the biosurfactant serrawettin W1 by Serratia marcescens S-1 improves hydrocarbon degradation. Bioprocess Biosyst. Eng..

[120] Tripathi V., Gaur V.K., Dhiman N., Gautam K., Manickam N. (2020). Characterization and properties of the biosurfactant produced by PAH-degrading bacteria isolated from contaminated oily sludge environment. Environ. Sci. Pollut. Res. Int..

[121] Bertrand B., Martínez-Morales F., Trejo-Hernández M.R. (2017). Upgrading Laccase Production and Biochemical Properties: Strategies and Challenges. Biotechnol. Prog..

[122] Agarwal N., Solanki V.S., Gacem A., Hasan M.A., Pare B., Srivastava A., Singh A., Yadav V.K., Yadav K.K., Lee C. (2022). Bacterial Laccases as Biocatalysts for the Remediation of Environmental Toxic Pollutants: A Green and Eco-Friendly Approach—A Review. Water.

[123] Zerva A., Simić S., Topakas E., Nikodinovic-Runic J. (2019). Applications of microbial laccases: Patent review of the past decade (2009–2019). Catalysts.

[124] Wang L., Tan Y., Sun S., Zhou L., Wu G., Shao Y., Wang M., Xin Z. (2022). Improving Degradation of Polycyclic Aromatic Hydrocarbons by *Bacillus atrophaeus* Laccase Fused with *Vitreoscilla* Hemoglobin and a Novel Strong Promoter Replacement. Biology.

[125] Dai X., Lv J., Wei W., Guo S. (2021). Effects of adding laccase to bacterial consortia degrading heavy oil. Processes.

[126] Neifar M., Chouchane H., Mahjoubi M., Jaouani A., Cherif A. (2016). *Pseudomonas extremorientalis* BU118: A new salt-tolerant laccase-secreting bacterium with biotechnological potential in textile azo dye decolourization. 3 Biotech.

[127] Muthukumarasamy N.P., Jackson B., Joseph Raj A., Sevanan M. (2015). Production of Extracellular Laccase from *Bacillus subtilis* MTCC 2414 Using Agroresidues as a Potential Substrate. Biochem. Res. Int..

[128] Galai S., Limam F., Marzouki M.N. (2009). A new *Stenotrophomonas maltophilia* strain producing laccase. Use in decolorization of synthetics dyes. Appl. Biochem. Biotechnol..

[129] Ali N.S., Huang F., Qin W., Yang T.C. (2022). Identification and Characterization of a New Serratia proteamaculans Strain That Naturally Produces Significant Amount of Extracellular Laccase. Front. Microbiol..

[130] Hölker U., Dohse J., Höfer M. (2002). Extracellular laccases in ascomycetes Trichoderma atroviride and Trichoderma harzianum. Folia Microbiol..

[131] Balaji V., Arulazhagan P., Ebenezer P. (2014). Enzymatic bioremediation of polyaromatic hydrocarbons by fungal consortia enriched from petroleum contaminated soil and oil seeds. J. Environ. Biol..

[132] Zafra G., Cortés-Espinosa D.V. (2015). Biodegradation of polycyclic aromatic hydrocarbons by Trichoderma species: A mini review. Environ. Sci. Pollut. Res..

[133] Zehra A., Dubey M.K., Meena M., Aamir M., Patel C.B., Upadhyay R.S. (2018). Role of Penicillium Species in Bioremediation Processes.

[134] Kapoore R.V., Padmaperuma G., Maneein S., Vaidyanathan S. (2022). Co-culturing microbial consortia: Approaches for applications in biomanufacturing and bioprocessing. Crit. Rev. Biotechnol..

[135] Qian X., Chen L., Sui Y., Chen C., Zhang W., Zhou J., Dong W., Jiang M., Xin F., Ochsenreither K. (2020). Biotechnological potential and applications of microbial consortia. Biotechnol. Adv..

[136] Tripathi V., Gaur V.K., Thakur R.S., Patel D.K., Manickam N. (2023). Assessing the half-life and degradation kinetics of aliphatic and aromatic hydrocarbons by bacteria isolated from crude oil contaminated soil. Chemosphere.

[137] Nnabuife O.O., Ogbonna J.C., Anyanwu C., Ike A.C., Eze C.N., Enemuor S.C. (2022). Mixed bacterial consortium can hamper the efficient degradation of crude oil hydrocarbons. Arch. Microbiol..

[138] Khan S.R., Nirmal Kumar J.I., Nirmal Kumar R. (2015). Enzymatic Evaluation During Biodegradation of Kerosene and Diesel by Locally Isolated Fungi from Petroleum-Contaminated Soils of Western India. Soil Sediment Contam..

[139] Vipotnik Z., Michelin M., Tavares T. (2021). Ligninolytic enzymes production during polycyclic aromatic hydrocarbons degradation: Effect of soil pH, soil amendments and fungal co-cultivation. Biodegradation.

[140] Boonchan S., Britz M.L., Stanley G.A. (2000). Degradation and mineralization of high-molecular-weight polycyclic aromatic hydrocarbons by defined fungal-bacterial cocultures. Appl. Environ. Microbiol..

[141] Zafra G., Absalón Á.E., Cuevas M.D.C., Cortés-Espinosa D.V. (2014). Isolation and selection of a highly tolerant microbial consortium with potential for PAH biodegradation from heavy crude oil-contaminated soils. Water. Air. Soil Pollut..

