# Supplementary material for: Exploring Novel Fungal–Bacterial Consortia for Enhanced Petroleum Hydrocarbon Degradation
Source: Toxics. 2024 Dec 17;12(12):913. doi: 10.3390/toxics12120913 (PMC11728489; doi:10.3390/toxics12120913)
Supplement: Supplementary file 1 [file toxics-12-00913-s001.zip › Supplementary Table S1.pdf]

Supplementary Table S1. Bacterial laccase activity over time.

Days	*Bacillus cereus* P05R8		*Burkholderia cepacia* P05R9		*Stenotrophomonas maltophilia* P05R11		*Burkholderia* sp. P05R16		*Serratia marcescens* P05R19	
	U/mL	D.O (600 nm)	U/mL	D.O (600 nm)	U/mL	D.O (600 nm)	U/mL	D.O (600 nm)	U/mL	D.O (600 nm)
0	0.000	0.00	0.000	0.00	0.000	0.00	0.000	0.00	0.000	0.00
1	0.583	1.09	0.638	0.90	0.916	1.54	0.555	0.11	0.694	1.64
2	0.444	2.12	0.361	1.88	0.361	1.50	0.500	0.79	0.305	1.80
3	0.027	1.34	0.000	1.12	0.027	1.19	0.055	1.04	0.083	1.76
4	0.388	1.12	0.305	0.87	0.305	1.10	0.333	0.81	0.361	1.65
5	0.305	1.08	0.333	0.79	0.222	1.03	0.194	0.62	0.361	1.46
6	0.277	1.05	0.333	0.80	0.250	0.91	0.194	0.64	0.140	1.40
