# Supplementary material for: Exploring Novel Fungal–Bacterial Consortia for Enhanced Petroleum Hydrocarbon Degradation
Source: Toxics. 2024 Dec 17;12(12):913. doi: 10.3390/toxics12120913 (PMC11728489; doi:10.3390/toxics12120913)
Supplement: Supplementary file 1 [file toxics-12-00913-s001.zip › Supplementary Table S2.pdf]

**Supplementary Table S2.** Fungal laccase activity over time.

| Days | *Penicillium janthinellum P05R1* | *Trichoderma koningiopsis P05R2* | *Penicillium janthinellum P05R3* | *Penicillium sp. P10R5* |
|---|---|---|---|---|
| | U/mL | U/mL | U/mL | U/mL |
| 0 | 0.000 | 0.000 | 0.000 | 0.000 |
| 1 | 0.000 | 0.000 | 0.000 | 0.000 |
| 2 | 0.000 | 0.000 | 0.000 | 0.000 |
| 3 | 0.000 | 0.000 | 0.167 | 0.000 |
| 4 | 0.049 | 0.056 | 0.167 | 0.000 |
| 5 | 0.000 | 0.130 | 0.161 | 0.000 |
| 6 | 0.000 | 0.148 | 0.167 | 0.000 |
| 7 | 0.000 | 0.278 | 0.241 | 0.000 |
| 8 | 0.000 | 0.222 | 0.278 | 0.000 |
| 9 | 0.000 | 0.185 | 0.296 | 0.000 |
| 10 | 0.167 | 0.250 | 0.278 | 0.000 |
| 11 | 0.111 | 0.333 | 0.167 | 0.083 |
| 12 | 0.194 | 0.296 | 0.278 | 0.111 |
| 13 | 0.203 | 0.425 | 0.240 | 0.111 |
| 14 | 0.250 | 0.000 | 0.204 | 0.111 |
