# Supplementary figures and images for: Exploring Novel Fungal–Bacterial Consortia for Enhanced Petroleum Hydrocarbon Degradation
Source: Toxics. 2024 Dec 17;12(12):913. doi: 10.3390/toxics12120913 (PMC11728489; doi:10.3390/toxics12120913)

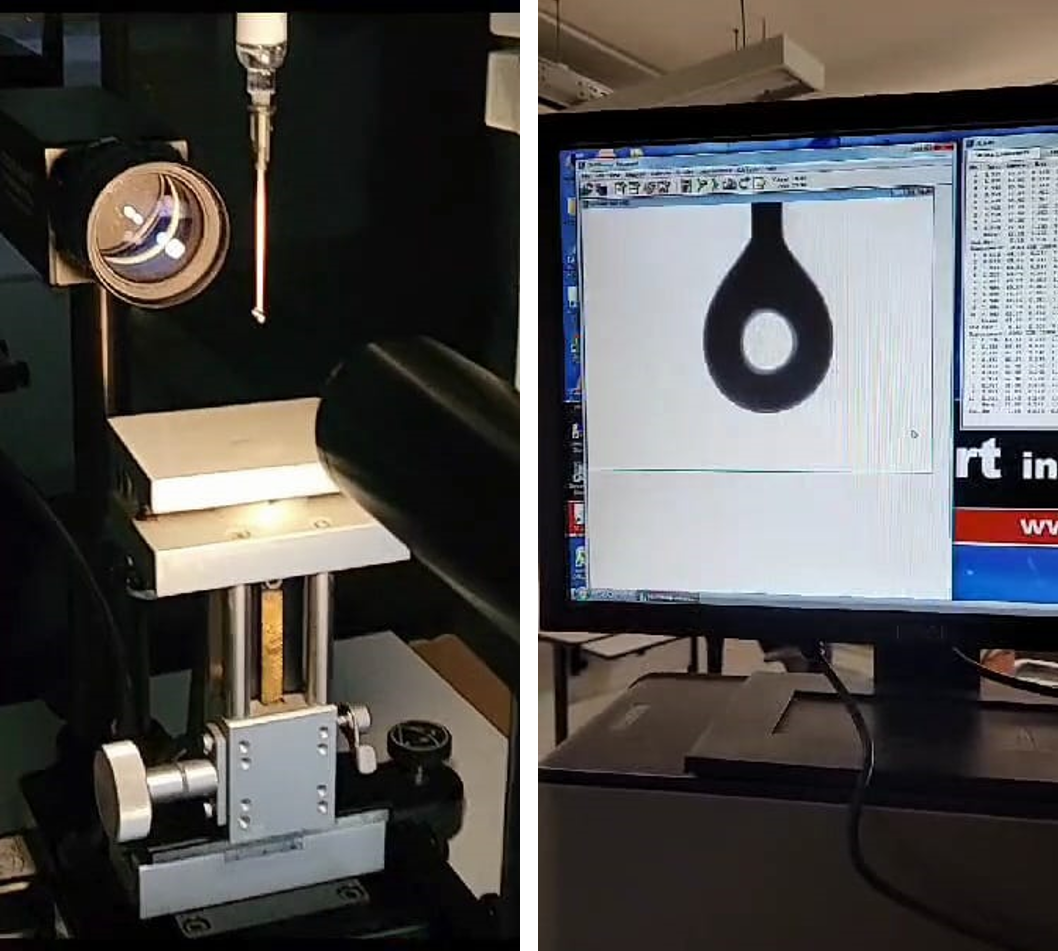

Supplement: Supplementary file 1 [file toxics-12-00913-s001.zip › Supplementary Figure S1.tif]

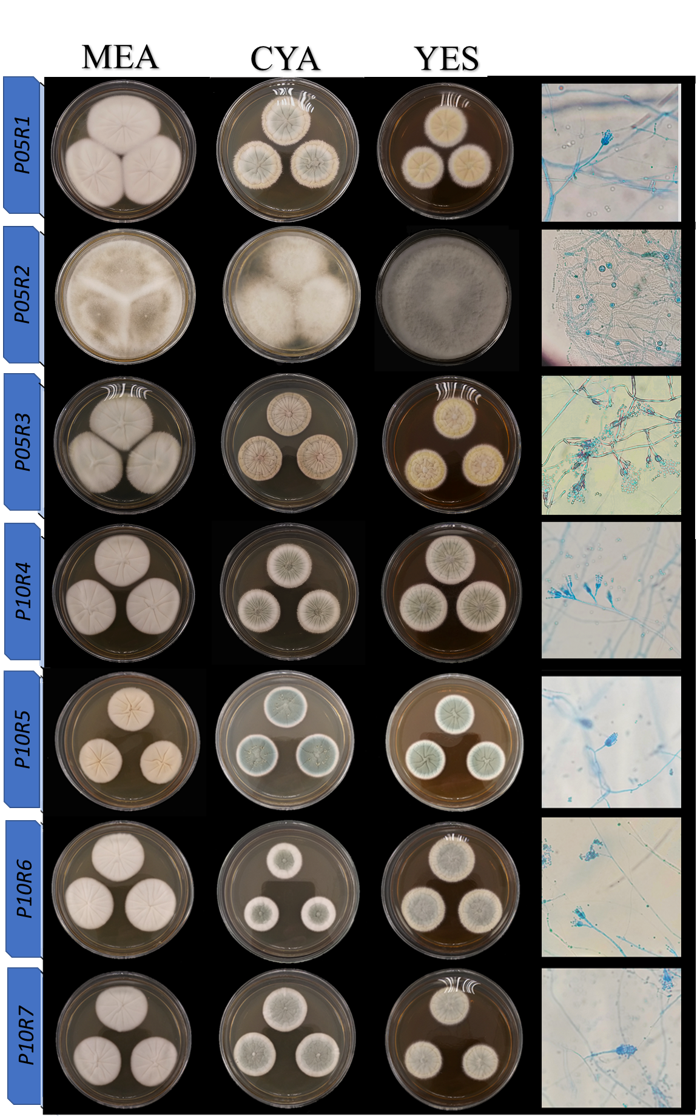

Supplement: Supplementary file 1 [file toxics-12-00913-s001.zip › Supplementary Figure S2.tif]
